# Carborane‐Induced Excimer Emission of Severely Twisted Bis‐*o*‐Carboranyl Chrysene

**DOI:** 10.1002/anie.201805967

**Published:** 2018-07-12

**Authors:** Adam V. Marsh, Nathan J. Cheetham, Mark Little, Matthew Dyson, Andrew J. P. White, Peter Beavis, Colin N. Warriner, Anthony C. Swain, Paul N. Stavrinou, Martin Heeney

**Affiliations:** ^1^ Department of Chemistry and Centre for Plastic Electronics Imperial College London London SW7 2AZ UK; ^2^ Department of Engineering Science University of Oxford Oxford OX1 3PJ UK; ^3^ Department of Physics and Centre for Plastics Electronics Imperial College London UK; ^4^ Molecular Materials and Nanosystems and Institute for Complex Molecular Systems Eindhoven University of Technology 5600 MB Eindhoven The Netherlands; ^5^ Department of Chemistry Imperial College London UK; ^6^ AWE Aldermaston, Reading RG7 4PR UK

**Keywords:** aggregation-induced emission, carboranes, chrysene, excimers, intramolecular charge transfer

## Abstract

The synthesis of a highly twisted chrysene derivative incorporating two electron deficient *o*‐carboranyl groups is reported. The molecule exhibits a complex, excitation‐dependent photoluminescence, including aggregation‐induced emission (AIE) with good quantum efficiency and an exceptionally long singlet excited state lifetime. Through a combination of detailed optical studies and theoretical calculations, the excited state species are identified, including an unusual excimer induced by the presence of *o*‐carborane. This is the first time that *o*‐carborane has been shown to induce excimer formation ab initio, as well as the first observation of excimer emission by a chrysene‐based small molecule in solution. Bis‐*o*‐carboranyl chrysene is thus an initial member of a new family of *o*‐carboranyl phenacenes exhibiting a novel architecture for highly‐efficient multi‐luminescent fluorophores.

At first glance, bulky boron‐rich spheroids appear to be an anathema to the two‐dimensional, π‐conjugated world of organic electronics. On the contrary, motivated by their unique three‐dimensional σ‐delocalization, electron‐withdrawing ability (when substituted at the carbon vertices),^1^ and high thermal and chemical stability, carboranes, and principally *o*‐carborane (C_2_B_10_H_12_), have now been incorporated in a wide range of molecular and polymeric semiconductors.^2^, ^3^, ^4^, ^5^, ^6^, ^7^, ^8^, ^9^, ^10^, ^11^, ^12^, ^13^, ^14^, ^15^, ^16^, ^17^, ^18^, ^19^, ^20^, ^21^, ^22^, ^23^, ^24^

A key property of *o*‐carborane is its ability to accept an excited charge via intramolecular charge transfer (ICT) from an aryl donor group, occurring most efficiently when the carborane has its C−C bond perpendicular to the aromatic plane. These ICT states are notable for their flexible carboranyl C−C bond, which can vibrate and relax non‐radiatively, or radiatively with a highly red‐shifted emission.^24^, ^25^, ^26^, ^27^, ^28^, ^29^, ^30^ Such systems often exhibit aggregation‐induced emission (AIE),^13^, ^14^, ^15^, ^17^, ^18^, ^19^, ^20^, ^22^, ^31^, ^32^ that is, in an aggregated state where molecular motion is restricted, non‐radiative relaxation pathways from the ICT state are inhibited, leading to a significant increase in emission efficiency. It has been demonstrated that incorporation of bulky carboranes on the long edges of linear acenes creates a large steric hinderance between the carboranes and proximal protons, leading to a considerable deformation of the ring system.^15^, ^16^, ^17^ With this comes a large increase in the carborane rotation barrier, and generally a more rigid structure; a property known to be useful for AIE.

*o*‐Carborane has also been shown, to a very limited extent, to aid excimer emission. When di‐substituted with two naphthyl donor groups, the imposed proximity of the aryl systems allows for an overlap of the aromatic wavefunctions, such that dimerization occurs readily when one naphthalene is in the excited state.^27^ In other cases where *o*‐carborane has been attached to pyrene, the bulky carborane moiety was found not to inhibit the π–π stacking required for pyrene excimer formation.^19^, ^20^, ^23^ In these few cases, however, the carborane can be considered essentially electronically isolated from the aromatic excimer species, save for some electron‐withdrawing inductive effects.

To explore whether *o*‐carborane could induce excimer emission ab initio, we focused our attention on chrysene, a non‐linear phenacene fluorophore. Chrysene is notable amongst polycyclic aromatic hydrocarbons for its lack of excimer emission. For decades it was thought chrysene could only form excimers in the solid state, following morphology changes at very high pressure.^33^, ^34^ Only recently have these excimers been observed in solution, requiring supramolecular DNA scaffolds to force chrysene units sufficiently close together in a face‐to‐face orientation such that excimer formation can occur.^35^

Inspired by these results, we planned to incorporate phenyl‐*o*‐carborane into the bay area of chrysene, theorizing that the bulky carborane would cause aromatic deformation to such an extent that π‐delocalization would be inhibited. As a consequence, the intermolecular repulsion potential would be lowered, conceivably allowing chrysene monomers to approach one another and support dimer formation.^33^ Also, we proposed that the π–π interactions between the phenyl group of the carborane and chrysene would help to lock the carboranyl C−C bond perpendicular to the aromatic plane in a geometry favorable for ICT, and in doing so create a new class of severely twisted, multi‐emissive luminescent materials.^36^

Herein, we present the synthesis of bis(phenyl‐*o*‐carborane)chrysene. We show through single‐crystal analysis the unique structure of this compound, including by far the largest α and β deformation angles reported to date for carboranyl donor–acceptor systems. A host of photoluminescence properties are displayed, which include localized emission (LE), ICT, and AIE, and excimer emission. Significantly, this is the first reported excimer of a chrysene based small molecule in solution, but most remarkably, it is the first example of an excimer in an *o*‐carborane containing molecule where the carborane is electronically involved, via a charge transfer state, in the dimerized species.

Scheme 1 shows the synthesis of 4,10‐bis(2‐phenyl‐1,2‐dicarbadodecaboran‐1‐yl)chrysene, **2**, from chrysene precursor **1**, itself prepared by copper‐free Sonogashira conditions from 4,10‐dichlorochrysene.^37^, ^38^ The decaborane coupling was first achieved using Bmim(Cl) as a catalyst,^39^, ^40^, ^41^ however the yield was discouragingly low (<2 %). A shift to the AgNO_3_ mediated decaborane–acetonitrile coupling afforded a more agreeable yield of 11 %.^42^ The main by‐products of these reactions were various non‐*closo*‐carboranyl chrysenes, suggesting mechanistically that the cage closure step did not proceed efficiently, likely as a result of the severely congested environment around the alkyne, resulting in relatively low yields.

**Scheme 1:**
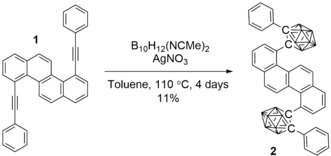
Synthesis of 4,10‐bis(2‐phenyl‐1,2‐dicarbadodecaboran‐1‐yl)chrysene (**2**).

The structure of **2** was elucidated by X‐ray diffraction of a single crystal grown from slow evaporation from a biphasic mixture of dichloromethane and hexane (Figure 1).

**Figure 1:**
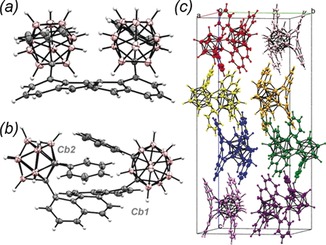
Structure of **2** a) along the chrysene short axis, b) long axis, and c) crystal packing, colored for clarity. Carboranes are discriminated by the labels Cb1 and Cb2.^53^

The incorporation of the carborane results in significant distortion of the usually planar chrysene core, affording a highly asymmetrically distorted ring system (compare the planar chrysene core of **1**, Supporting Information, Figures S8–10). Borrowing from the deformation parameters of anthracene‐based molecules, α and β, and adding a new parameter γ, the distortion of **2** is summarized in the Supporting Information, Figure S11. The carboranes are displaced out of the aromatic plane to a degree much larger than other recently reported carborane‐containing aromatic systems (Supporting Information, Figure S12).^15^, ^16^, ^17^ The γ parameter highlights further distortion, with the carboranes shifted perpendicular to the plane of the rings and also parallel to them, away from the bay‐area protons and the *m*‐edge C−C bond.

The severely twisted geometry induced by the bulky carboranes results in a complex array of emissive species, as revealed by photoluminescence emission (PL) measurements, shown in Figure 2.

**Figure 2:**
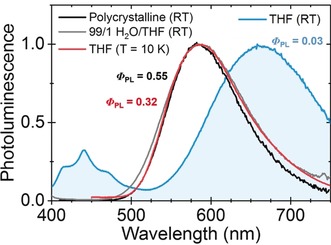
Normalized PL spectra of **2** in THF (*λ*_ex_=390 nm), in 99/1 v/v H_2_O:THF (*λ*_ex_=390 nm), in the solid‐state (*λ*_ex_=385 nm), and in THF at low temperature (10 K, *λ*_ex_=400 nm). All solution concentrations ca. 10^−4^ m.

In the THF solution of **2** at RT, a dual emissive profile is observed, with a vibronic emission band centered at 450 nm and a long‐wavelength, broad, featureless peak around 660 nm. The emission from polycrystalline, 99/1 H_2_O/THF and THF (*T*=10 K) aggregated samples of **2**,^43^ are all dominated by a common peak around 580 nm, also featureless and highly red‐shifted in comparison to the ground state absorption. For samples when **2** is in an aggregated state, the overall emission efficiency is significantly higher (ca. ×10) with recorded efficiencies (PLQE) *Φ*_PL_=0.03, 0.32 and 0.55 for THF (RT) solution, 99/1 H_2_O/THF solutions and polycrystalline samples, respectively. The high‐efficiency, 580 nm peak is hence firmly associated with an AIE process.

The vibronic progression around 450 nm is characteristic of LE originating from chrysene,^44^ but curiously, for the THF solution, the LE spectrum of **2** shows some excitation dependence (Supporting Information, Figure S13). While the different emission efficiencies offer some insight into the long‐wavelength peaks shown in Figure 2, their distinct natures are not immediately apparent from the PL spectra. Monitoring photoluminescence intensity at the emission peaks, that is, at 580 nm (AIE) and 660 nm, by photoluminescence excitation (PLE) spectroscopy (Supporting Information, Figures S14 and S15) reveals a commonality between the two peaks, faithfully mirroring the ground state absorption profile obtained from UV/Vis spectroscopy (Supporting Information, Figures S16–S20), spectrally similar to that expected from chrysene.^45^ The results suggest a geometry rearrangement is required post‐absorption for these emissions to occur, consistent with both ICT, where the carborane C−C bond elongates after accepting an excited charge,^25^ and excimer formation. Both mechanisms are also consistent with the large Stokes shift observed. Time‐resolved measurements (Supporting Information, Figures S21 and S22) highlight further differences. The lifetimes of the aggregated solution (*τ*=23 ns; *λ*_em_=580 nm) and film (*τ*=17 ns; *λ*_em_=580 nm) are significantly longer to those found in the THF solution (*τ*=0.18 and 3.1 ns; *λ*_em_=436 and 657 nm, respectively); indeed a lifetime of 23 ns is extremely long‐lived for singlet emission from an organic fluorophore.^46^ Similar long lifetimes have been observed for other carborane‐containing AIE‐active compounds for which aggregated emissions are assigned to ICT species.^15^, ^16^, ^17^, ^18^, ^19^, ^20^

To further probe the character of the long wavelength emissions, a temperature dependent PL study was undertaken (Figure 3). At room temperature the solution of **2** in CHCl_3_ is dual‐emissive, and the emissive profile shows little change until the freezing point of CHCl_3_ is reached (ca. 210 K). Upon decreasing the temperature towards the solvent freezing point, the long wavelength peak red‐shifts and reduces in intensity. In contrast, and for temperatures below the solvent freezing point, the trend has reversed, revealing a definite blue‐shift along with a significant increase of the AIE peak. Furthermore, upon freezing (*T*=210–150 K), a notable increase in peak width is initially observed and consistent with the co‐existence of two emission species (580 nm and 660 nm) as a fraction of **2** is educed out of the solution, (Supporting Information, Figure S24). Similar observations are found in a variable temperature study of **2** in THF (Supporting Information, Figure S25), and in a H_2_O/THF solvent composition study (Supporting Information, Figure S26). The sharp change in emission profile seen around the solvent freezing point in the former is mirrored at an approximate 50/50 H_2_O/THF composition in the latter. The transition is also easily detected with the naked eye (Supporting Information, Figure S27). From this data, we infer that the 580 nm AIE peak is the result of a common excited‐state species present in all samples, and significantly, in contrast to similar carboranyl donor–acceptor compounds,^15^, ^16^, ^18^ it appears to be distinct in origin to the long wavelength emission observed in good solvents.

**Figure 3:**
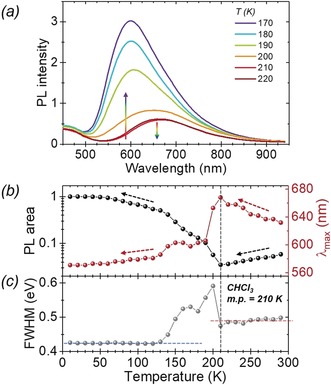
Photoluminescence emission of **2** (a) in CHCl_3_ (*λ*_ex_=400 nm) as a function of temperature; b) PL area of long wavelength to LE ratio and long wavelength emission *λ*_max_; c) full width half maximum (FWHM) as a function of temperature. Concentration ca. 10^−4^ m.

The excited state character can be investigated by solvatochromism with the aid of a Lippert–Mataga plot, as shown in the Supporting Information, Figure S31.^47^ The samples of **2** in good solvents, with Hildebrand solubility parameters around 9 cal^1/2^ cm^−3/2^, follow a positive linear trend in Stokes shift with the Hansen polarity parameter for the long wavelength peak around 660 nm,^48^ characteristic of a charge‐transfer nature. Poor solvent solutions of **2** do not conform to this trend, which is most likely due to the occurrence of local aggregation.^49^ For these cases, it is possible there is no longer one dominant emission species, rather two similarly contributing and competing emissions as evidenced by broadened emission peaks. This is akin to the PL profile exhibited by the 50/50 H_2_O/THF solution composition of **2**; however, without observing the dynamic change between the AIE and 660 nm peaks, we cannot wholly discount the presence of other emissive species. Nevertheless, the study clearly demonstrates the 660 nm emission has a strong delocalized, charge‐transfer character. Finally, we note the Stokes shift for the LE emission is virtually independent of solvent quality, further supporting this is indeed emission originating from the chrysene core.

To probe the role, if any, of excimers, Figure 4 outlines the effect of varying concentration on the emission of **2** in THF. Of interest here is the stark reduction in the 660 nm peak, relative to the LE peak, below a critical concentration of about 10^−6^ m. At a concentration of 10^−8^ m, the 660 nm peak has all but disappeared, while a small amount of blue‐shifted emission (Figure 4 b) remains. We attribute this to the 580 nm emissive species, which appears to be present in tiny amounts (Supporting Information, Figure S13). The 660 nm emission is consistent with an excited state dimerization process where, above a critical concentration (ca. 10^−6^ m), emission from the excimer dominates, whilst below this most excited state relaxation occurs prior to excimer formation. On this basis and supported by evidence from the PL and solvatochromism study, we attribute the 660 nm emission to an excimer species formed from a ground state and an excited molecule of **2**; this is the first time excimer emission has been observed in a chrysene‐based small molecule.^33^, ^35^, ^44^

**Figure 4:**
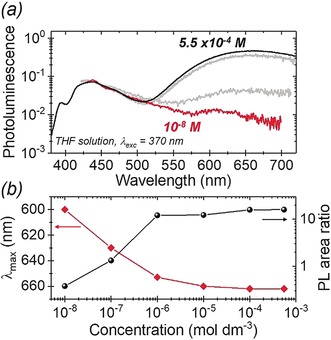
Concentration‐dependent emission of **2** in THF: a) normalized PL spectra following 370 nm excitation, and b) *λ*_max_ of long wavelength peak and long wavelength to LE PL area ratio, as a function of concentration.

The novelty and significance of this excimer can be further appreciated by comparing the degree of Stokes shift from the peak emission (*λ*_max_=660 nm) of **2** with that of the pristine chrysene excimer (*λ*_max_=475 nm).^35^ Given the severe twisting of the chrysene backbone and steric hindrance induced by carboranyl groups, the excimer of **2** is distinct to that previously observed, not solely originating from a dimer of the chrysene core. Rather, the lower energy emission points to a more electronically delocalized excimer species, consistent with dimerization where at least one of the molecules exhibits ICT character, with delocalization across the chrysene core onto the carboranyl groups.

The presence of the 580 nm peak even at very low concentrations suggests a unimolecular excited state is responsible for this emission. To investigate this, a theoretical study employing density functional theory (DFT) and time‐dimensional DFT (TD‐DFT) was performed, detailed in SI Experimental. Emission wavelengths were calculated, as a function of the relative dihedral angles (*ϕ*) of Cb1 and Cb2 following vertical excitation with solvent relaxation, and presented in full in the Supporting Information, Figures S33 and S35, with selected data shown in Figure 5. The first point of note is that, except “Cb1 (*ϕ*), Cb2(*ϕ*)” of “0°, 0°”, all geometries exhibit localization of electron density onto the chrysene core as indicated by the frontier molecular orbitals (MOs) of the S_1_ excited state. The calculated emission wavelengths of these all span the range of 296–500 nm, in good agreement with observed LE PL emission. It appears that, depending on the relative dihedral geometries of the carboranes, chrysene is twisted to varying degrees, leading to significant differences in absorption and emission energies, that is, higher degrees of twisting leads to shorter absorption and emissive wavelengths and vice versa.^50^ As such, the complexity of the vibronic region of **2** is explained by the flexing of the chrysene core as the carboranes rotate around *ϕ*. Secondly, the “0°, 0°” global minimum ground state geometry (with carborane C−C bonds virtually perpendicular to the aromatic plane) is the only geometry which shows a strong ICT character for the S_1_ excited state, that is, from the chrysene donor to Cb1. Accordingly, a significantly lower emissive energy is calculated in comparison with all other geometries, spectrally consistent with the observed AIE emission. Furthermore, a dihedral potential energy scan (PES) carried out in the crystal bulk using an ONIOM model shows a deeper potential energy well around the global minimum geometry, on the order of that of 1,1′‐binaphthol (BINOL), a molecule with a steric‐induced axis of chirality at room temperature.^51^ This suggests that in aggregated states the ground state geometry is significantly more favorable than other geometries, further favoring ICT emission.

**Figure 5:**
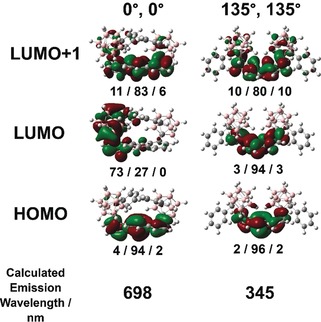
Selected DFT calculated S_1_ excited state geometries and molecular orbitals of **2** in THF with emission wavelengths and relative dihedral angles (*ϕ*) of Cb1 and Cb2, as specified in the Supporting Information, Figure S35. The relative molecular orbital contributions from Cb1/chrysene/Cb2 are displayed below each geometry.

We have sought to clarify two important points regarding the long‐wavelength emission species. First, given the ICT state of **2** involves a charge transfer from the chrysene to the carborane, it seems reasonable to expect the excimer of **2** has a similar ICT character. Remarkably, this is, to the best of our knowledge, the first example of an excimer species involving a carborane with ICT character. Secondly, and in line with many similar carborane‐containing fluorophores,^13^, ^14^, ^15^, ^17^, ^18^, ^19^, ^20^, ^22^, ^31^, ^32^ the AIE observed for **2** is the result of a unimolecular ICT species. These two points may also be used to rationalize the differences between the emission efficiencies recorded from the two long‐wavelength emissions. We believe that establishing these two distinct processes is a significant result, highlighting *o*‐carborane as a novel excimer‐inducible element block,^52^ and consequently, that ICT is not necessarily a sufficient explanation for long wavelength emission observed from carborane‐containing donor–acceptor systems.

In conclusion, we have synthesized a bis‐*o*‐carborane based chrysene derivative, the first member of a new class of severely twisted luminescent phenacene‐based carboranes, exhibiting the highest α and β deformation angles of any reported carborane‐containing compound. Optical studies revealed a complex, excitation dependent, multi‐emissive profile, demonstrating AIE with good quantum efficiency (*Φ*_PL_=0.32) and exceptionally long singlet excited state lifetimes (*τ*=23 ns). The nature of the two broad, featureless and highly red‐shifted emissions was initially ambiguous, but solvatochromism studies revealed the 660 nm peak to have significant charge‐transfer character and concentration studies confirmed the emissive species to be an excimer. While the origin of the AIE emissive species was not definitively confirmed, ICT is believed to be responsible. Significantly, this is the first reported excimer to electronically involve a carborane as well as the first solution‐based chrysene excimer not requiring a supramolecular scaffold. Through innovative architectural design, we were able to induce excimer formation and AIE by locking the molecule in a geometry highly favorable for ICT formation.

## Conflict of interest

The authors declare no conflict of interest.

## Supporting information

As a service to our authors and readers, this journal provides supporting information supplied by the authors. Such materials are peer reviewed and may be re‐organized for online delivery, but are not copy‐edited or typeset. Technical support issues arising from supporting information (other than missing files) should be addressed to the authors.

Supplementary
