# Supplementary material for: Carborane‐Induced Excimer Emission of Severely Twisted Bis‐o‐Carboranyl Chrysene
Source: Angew Chem Int Ed Engl. 2018 Jul 12;57(33):10640–5. doi: 10.1002/anie.201805967 (PMC6099267; doi:10.1002/anie.201805967)
Supplement: Supplementary file 1 — Supplementary [file ANIE-57-10640-s001.pdf]

Angewandte Chemie
Eine Zeitschrift der Gesellschaft Deutscher Chemiker

Supporting Information

Carborane-Induced Excimer Emission of Severely Twisted Bis-*o*-Carboranyl Chrysene

Adam V. Marsh, Nathan J. Cheetham, Mark Little, Matthew Dyson, Andrew J. P. White, Peter Beavis, Colin N. Warriner, Anthony C. Swain, Paul N. Stavrinou, and Martin Heeney**

anie_201805967_sm_miscellaneous_information.pdf

Table of Contents

Experimental Procedures 3
Synthesis 4
NMR of **1** and **2** 6
Mass and Infra-red spectrum of **2** 9
X-ray of **1** and **2** 10
Depiction of carborane induced deformation of chrysene 11
Depiction of deformation quantities of **2** 12
α and β values for literature carborane donor-acceptors 13
UV-Vis 14
PL and PLE 16
Time-Resolved PL 20
Summary of PLQE 21
Low Temperature-dependent PL 22
H_2O:THF solvent composition-dependent PL 24
Picture of the PL of **2** in various H_2O:THF compositions 25
High Temperature-dependent PL 26
Solvent-dependent PL 27
Computational calculations 29
Cyclic Voltammetry 32
References 33

Experimental Procedures

Dry solvents and reagents were purchased from Sigma Aldrich, VWR, Alfa Aesar, Acros organics or Apollo scientific and used without further purification, and all reactions were performed under BOC Pureshield Argon. Proton (^{1}H), boron (^{11}B) and carbon (^{13}C) solution state NMR spectra were obtained from a Bruker Avance-400 (400 MHz) spectrometer using $CDCl_3$ as the deuterated solvent unless otherwise stated. Chemical shift data was produced using residual solvent internal standards (^{1}H: $CHCl_3$ at 7.26 ppm; ^{13}C: $CDCl_3$ at 77.36 ppm). Mass spectrometry was carried out on an Agilent HP6890 GC (EI); and ultrafleXtreme (MALDI-ToF). Infra-red spectroscopy measurements were obtained using a PerkinElmer FT-IR Spectrum 100 fitted with an ATR (Ge/Ge) accessory. X-ray crystallographic data was obtained at 173 K, using an Agilent Xcalibur PX Ultra A. Cyclic Voltammetry was carried out using a Metrohm Autolab PGSTAT101 potentiostat in dry DCM containing 0.1 M NBu_4PF_6 with a platinum working electrode, Pt counter electrode and an Ag/AgCl (Ag/Ag^+) reference electrode with ferrocene as internal reference. UV spectra were taken using a Shimadzu UV-1800 UV spectrometer.

PL: Steady-state photoluminescence emission and excitation measurements were peformed using a Horiba Scientific FluoroMax 4 spectrofluorometer. Solution measurements were performed using 1 cm path length quartz cuvettes. Solid-state photoluminescence and photoluminesence quantum efficiency (PLQE) measurements were performed in the Horiba Quanta-Phi diffusely-reflecting integrating sphere attachment. PLQE was calculated using the methodology detailed by Ahn et al.[1] The setup was calibrated by testing fluorescence standards Rhodamine 6G and Fluorescein to ensure the accuracy and reliability of calculated PLQE values.

Solution time-resolved PL: Solution time-resolved photoluminescence measurements were performed on THF and 99/1 H_2O/THF v/v ratio solutions in 1 cm quartz cuvettes. The excitation source was a 379 nm laser excitation source, pulsed at 10 MHz and focused onto the solution with a x20 microscope objective. Resulting photoluminescence was focused into a Chromex 250 IS imaging spectrograph coupled to a Hamamatsu C4334 Streakscope detector. The time-range of the measurement was 20 and 200 ns for the THF and 99/1 H_2O/THF solutions respectively. The streak-camera setup allowed for collection of full emission spectra (400-680 nm, 3 nm spectral resolution) at every time point, which were subsequently integrated as a function of time to obtain PL kinetic traces of different emission regions.

Film time-resolved PL: Thin films were prepared by spin-coating a 5 mg mL^{-1} chlorobenzene solution of **2** on VWR Plain Micro Slides using a Laurell Model WS-650-23 spin coater under nitrogen atmosphere, with a 1000 rpm spin speed and 2 min spin time. Film time-resolved photoluminescence measurements were carried out on a Horiba Scientific DeltaFlex TCSPC system, with a PPD 650 picosecond photon detection module and a IBH NanoLED 404 nm pulsed diode excitation source. The time-range of the measurement was 100 ns, and PL kinetic traces were collected at 580 and 650 nm with 2 nm spectral resolution.

Temperature dependent photoluminescence measurements were performed on **2** in solution, held in a 1 mm path length fused silica cuvette. Samples were inside a helium filled gas cooled closed-cycle cryostat, with spectra recorded at 10 K increments. The sample temperature was held constant via for 5 minutes prior to each measurement, enabling the sample to reach thermal equilibrium. The excitation wavelength was 400 nm, produced using a monochromated supercontinuum light source, with a long pass filter at 450 nm to attenuate the scattered excitation beam. Emitted light was collected perpendicular to excitation and focused into a 100 µm diameter optical fibre. An Andor SR-163 spectrometer was then used to disperse the emitted light onto a CCD (Andor i-Dus), with the signal averaged over 50 accumulations of 0.1 s exposure to reduce noise. A dark background was subtracted from all spectra before correcting with a calibration file derived from a known light source to account for both detector response and that of the long pass filter. No changes aside from temperature were made to the optical configuration between measurements.

Computational studies were carried out using Gaussian G09 rev. d01 and GaussView 5.0.9 visualization software.[2] DFT calculations were accomplished using an ω tuned ωB97XD functional, with the tuning performed according to the methodology described by Körzdörfer and Brédas, and a 6-31G(d) basis set.[3] Molecular mechanics (MM) calculations were carried out using a universal force field (UFF) model. Solvent was modelled using a self-consistent reaction field (SCRF) approach using the polarisable continuum model. For solid state calculations a multilayer ONIOM model was used; high-level was treated with DFT and low-level with MM, with electronic embedding. Where appropriate, energy minima were identified following geometry optimisation and frequency calculations returning zero imaginary frequencies.

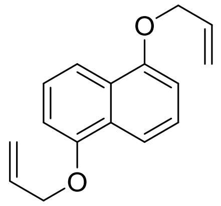

1,5-Bis(allyloxy)naphthalene

In a modification to a literature procedure,[4] allyl bromide (32.19 mL, 375 mmol) was added to a suspension of 1,5 dihydroxynaphthalene (25.00 g, 156 mmol) and K_2CO_3 (51.75 g, 375 mmol) in dry acetonitrile (400 mL) and stirred overnight at room temperature. The inorganic components were separated by vacuum filtration and washed with diethyl ether (300 mL), the filtrate was washed with water (5 x 200 mL), brine (2 x 200 mL) and dried over $MgSO_4$. The solution was concentrated in vacuo and poured into methanol (300 mL) cooled to -78 °C. The precipitate was collected, washed with methanol and dried yielding the *title compound* as a light brown solid (24.46 g, 65%). MP 90-92 °C. ^{1}H NMR (400 MHz, $CDCl_3$) δ 7.90 (2H, d, *J* = 8.6 Hz), 7.36 (2H, dd, *J* = 8.6, 7.7 Hz), 6.85 (2H, d, *J* = 7.7 Hz), 6.18 (2H, ddt, *J* = 17.2, 10.6, 5.0 Hz), 5.52 (2H, dq, *J* = 17.2, 1.6 Hz), 5.34 (2H, dq, *J* = 10.6, 1.6 Hz), 4.72 (4H, dt, *J* = 5.0, 1.6 Hz) ppm. ^{13}C NMR (100 MHz, $CDCl_3$) δ 154.5, 133.7, 127.2, 117.7, 114.9, 106.2, 69.3 ppm. HRMS (EI$^+$) $C_{16}H_{16}O_2$ requires 240.1150, found 240.1153.

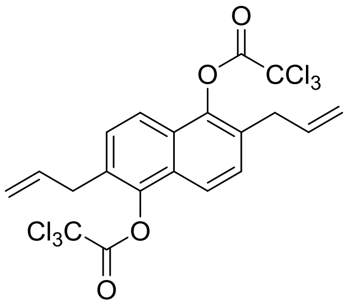

2,6-Diallyl-1,5-bis(2,2,2-trichloroacetyl)naphthalene

In a modification to a literature procedure,[4] 1,5-Bis(allyloxy)naphthalene (15.00 g, 62.5 mmol) was heated in an inert atmosphere, with stirring, for 2 h at 210 °C, after which the heating was removed and the orange-brown solid was allowed to cool to room temperature. Dry diethyl ether (300 mL) and pyridine (12.00 mL, 150 mmol) were added, the solution was cooled to 0 °C and trichloroacetyl chloride (16.74 mL, 150 mmol) was added dropwise. The mixture was stirred for 2 h then allowed to warm to room temperature. The brown solution was poured into a slurry of ice-water (200 g) and $NaHCO_3$ (5 g), washed with water (2 x 100 mL), saturated aq. NH_4Cl (3 x 100 mL), water (3 x 100 mL), brine (2 x 100 mL), and dried over $MgSO_4$. Solvent was removed in vacuo yielding the *title compound* as a yellow-brown solid (27.23 g, 82%) which was subsequently used without further purification. MP 132-133 °C. ^{1}H NMR (400 MHz, $CDCl_3$) δ 7.82 (2H, d, *J* = 8.6 Hz), 7.49 (2H, d, *J* = 8.6 Hz), 6.01-5.85 (2H, m), 5.17-5.04 (4H, m), 3.55-3.48 (4H, m) ppm. ^{13}C NMR (100 MHz, $CDCl_3$) δ 160.6, 140.0, 134.9, 129.7, 120.2, 89.8, 34.4 ppm. HRMS (EI$^+$) $C_{20}H_{14}Cl_6O_4$ requires 527.9023, found 527.9045.

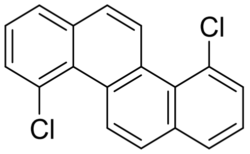

4,10-Dichlorochrysene

2,6-Diallyl-1,5-bis(2,2,2-trichloroacetyl)naphthalene (4.00 g, 7.53 mmol) was dissolved in dry diglyme (4 mL) and the solution was purged with argon for 20 mins. Cuprous chloride (5 mol%, 37 mg, 0.38 mmol) was added and the mixture was purged with argon for a further 20 mins before being heated to reflex for 2 h, or until HCl gas emission ceased. The mixture was cooled, loaded directly onto a flash chromatography column and eluted with 1:9 DCM:Pet. ether. The product fractions were left to slowly evaporate, yielding the *title compound* as light-gold microcrystals (604 mg, 27%). MP 159-161 °C. ^{1}H NMR (400 MHz, $CDCl_3$) δ 9.52 (2H, d, *J* = 9.1 Hz),7.90 (2H, dd, *J* = 7.8, 1.3 Hz), 7.85 (2H, d, *J* = 9.1 Hz), 7.76 (2H, dd, *J* = 7.8, 1.3Hz), 7.53 (2H, t, *J* = 7.8 Hz) ppm. ^{13}C NMR (100 MHz, $CDCl_3$) δ 134.9, 131.8, 130.1, 130.2, 127.9, 127.8, 126.9, 126.8, 125.3 ppm. HRMS (EI$^+$) $C_{18}H_{10}Cl_2$ requires 296.0160, found 296.0154.

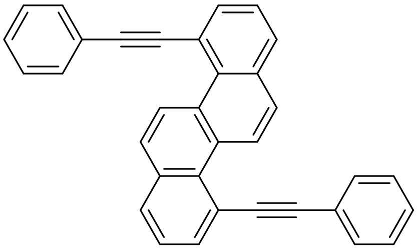

4,10-Bis(phenylethynyl)chrysene

4,10-Dichlorochrysene (500 mg, 1.68 mmol), cesium carbonate (0.55 g, 1.68 mmol), bis(triphenylphosphine)palladium(II) dichloride (10 mol%, 118 mg, 0.17 mmol) tri(cyclohexyl)phosphine (20 mol%, 96 mg, 0.34 mmol) and phenylacetylene (0.74 mL, 6.73 mmol) were added to a flask charged with dry DMF (7 mL), and the mixture was purged with argon before being heated to 110 °C overnight. After cooling to room temperature, the mixture was added directly to a silica plug (Pet. ether), which was eluted first with Pet. ether, then 1:5 DCM:Pet. ether to eluted the crude product. Solvent was removed in vacuo and the yellow oil was subjected to column chromatography with 1:19 DCM:Pet. ether, yielding the *title compound* as bright-green crystals (254 mg, 35%). Crystals suitable for x-ray diffraction analysis were grown by slow evaporation of a biphasic solution of DCM and hexane. MP 184 °C. ^{1}H NMR (400 MHz, $CDCl_3$) δ 10.30 (2H, d, *J* = 9.1 Hz) 8.05-7.96 (6H, m), 7.69 (4H, m), 7.62 (2H, td, *J* = 7.7, 0.4 Hz), 7.48-7.38 (6H, m) ppm. ^{13}C NMR (100 MHz, $CDCl_3$) δ 135.1, 133.4, 131.7, 130.6, 130.2, 129.7, 128.9, 128.8, 126.5, 126.0, 125.7, 124.1, 120.1, 95.2, 92.7 ppm. HRMS (EI^+) $C_{34}H_{20}$ requires 428.1565, found 428.1579.

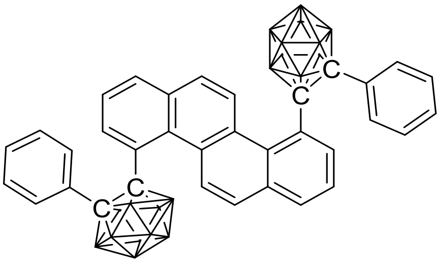

4,10-Bis(2-phenyl-1,2-dicarbadodecaboran-1-yl)chrysene

BmimCl method:

1 (250 mg, 0.58 mmol) and decaborane (138 mg, 1.13 mmol) were added to a vigorously stirring flask charged with a biphasic mixture of bmim(Cl) (59 mg, 0.34 mmol) and dry toluene (10 mL), and the mixture was heated to reflux for 72 h. After cooling the solvent was removed in vacuo and the crude orange oil was subjected to column chromatography with 1:19 DCM:Pet. ether, yielding **2** as bright yellow microcrystals (7 mg, 2%).

$AgNO_3$ method:

Decaborane (552 mg, 6.96 mmol) was dissolved in a stirring solution of dry CH_3CN (1 mL) and the mixture was heated to 60 °C for 1h. After cooling the mixture was diluted with dry toluene (10 mL), 4,10-bis(phenylethynyl)chrysene (**1**, 750 mg, 1.74 mmol) and $AgNO_3$ (14 mg) were added, and the mixture was heated to reflux for 4 days. After cooling the solvent was removed in vacuo and the crude orange oil was subjected to column chromatography with 1:19 DCM:Pet. ether, yielding **2** as bright yellow microcrystals (127 mg, 11%). Crystals suitable for x-ray diffraction analysis were grown by slow evaporation of a biphasic solution of DCM and hexane. MP >350 °C. ^{1}H NMR (400 MHz, $CDCl_3$) δ 8.45 (2H, d, *J* = 8.8 Hz), 8.35 (2H, dd, *J* = 7.8, 1.0 Hz), 7.48 (2H, dd, *J* = 7.8, 1.0 Hz), 7.36 (2H, t, *J* = 7.8 Hz), 7.19 (2H, ddt, *J* = 7.5, 7.5, 1.6 Hz) 7.09 (2H, d, *J* = 8.8 Hz), 7.00 (4H, ddt, *J* = 8.8, 7.5, 1.6 Hz), 6.77-6.72 (4H, m), 4.01-1.59 (20H, br) ppm. ^{11}B NMR (128 MHz, $CDCl_3$) δ -0.91, -2.14, -3.17, -6.80, -10.14 ppm. ^{13}C NMR (100 MHz, $CDCl_3$) δ 139.8, 132.6, 132.5, 131.8, 131.4, 131.3, 130.4, 130.4, 129.8, 128.3, 126.7, 124.8, 122.2, 90.5, 89.0 ppm. MS (MALDI-ToF) $C_{34}B_{20}H_{40}$ requires 665.5, found 665.5. HRMS in Figure S6. IR (ν cm^{-1}) 2565 (B-H).

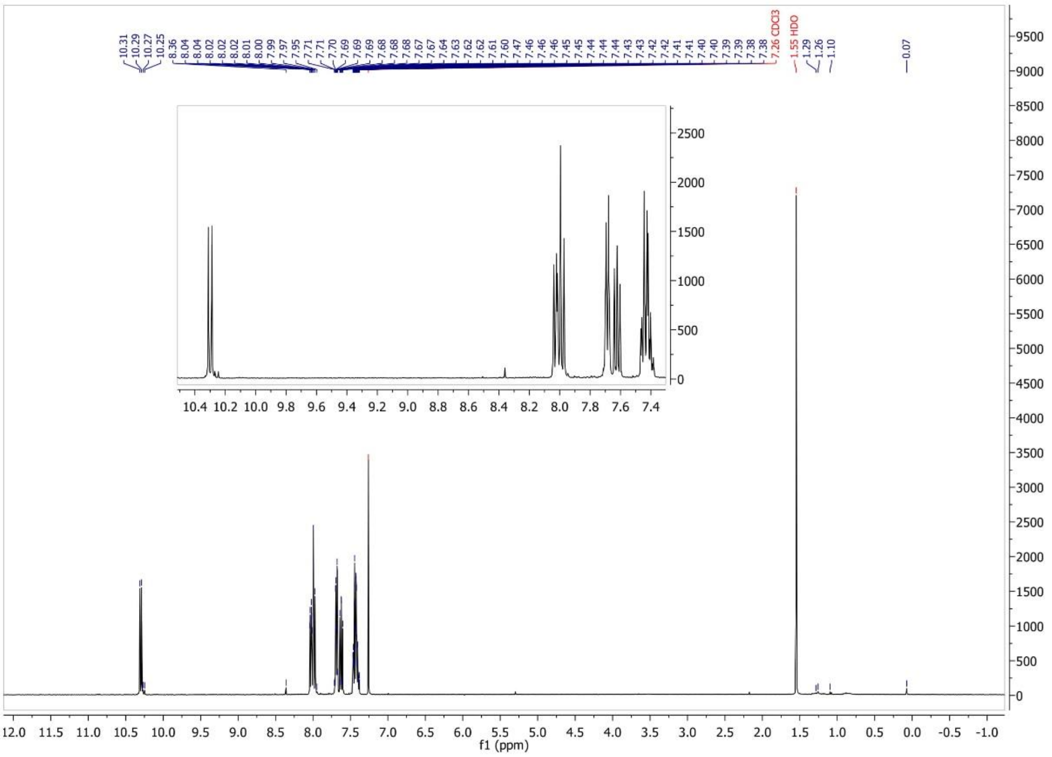

Figure S1. ^{1}H of **1** in $CDCl_3$.

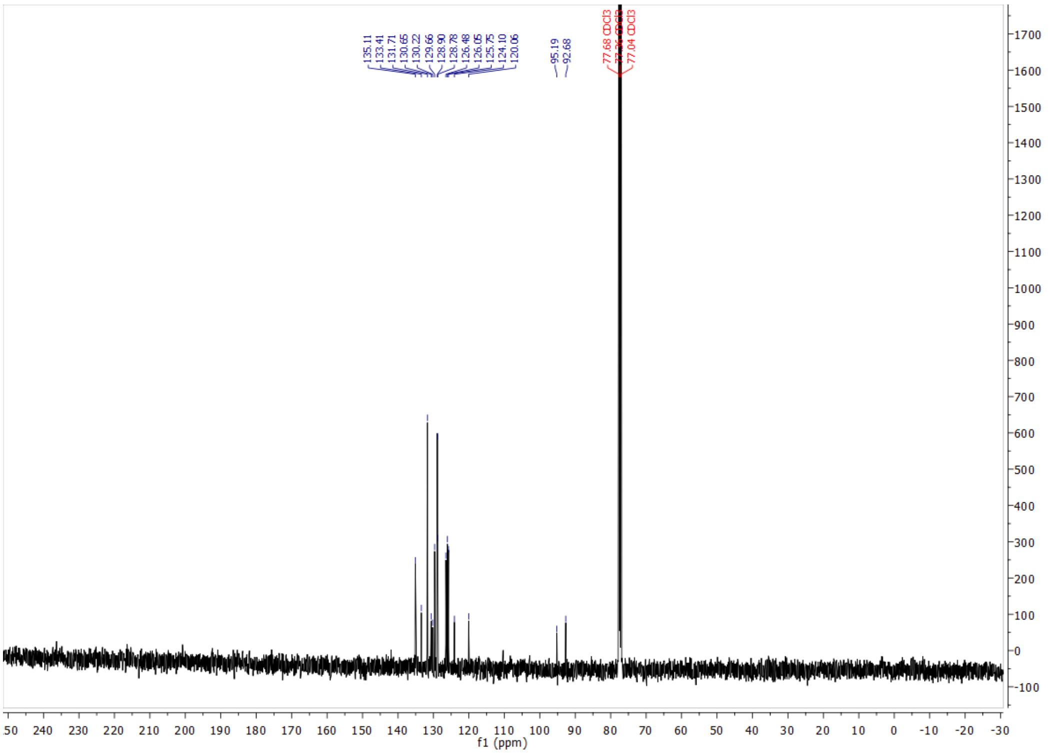

Figure S2. ^{13}C of **1** in $CDCl_3$.

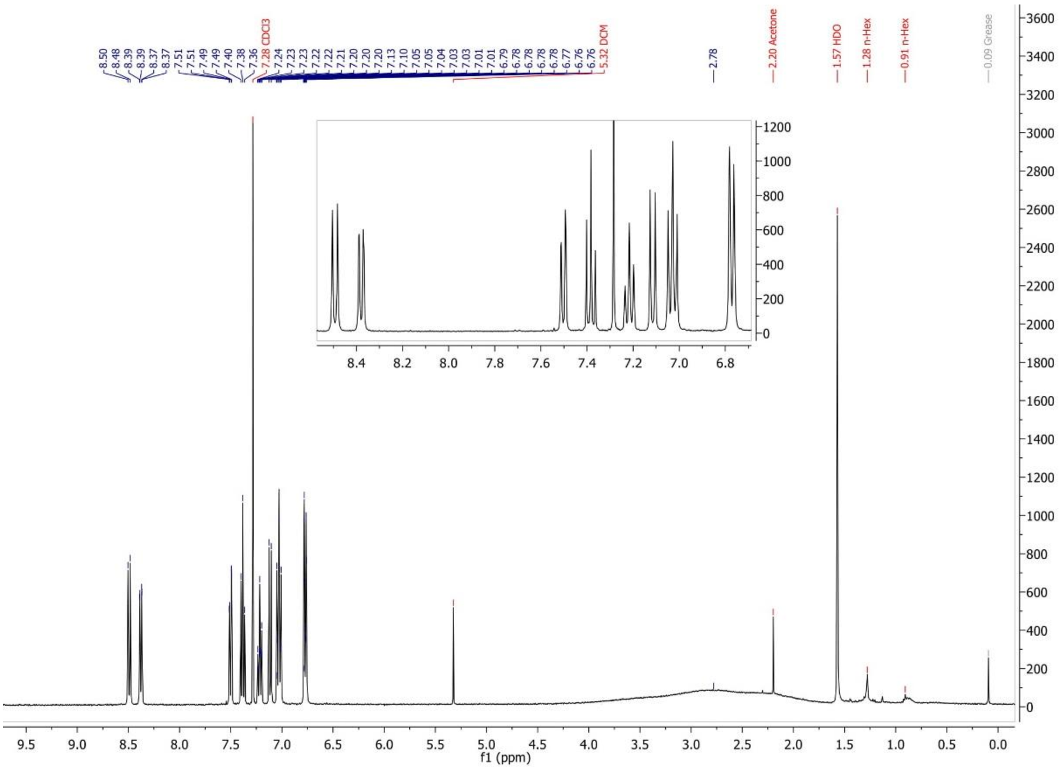

Figure S3. ^{1}H of **2** in $CDCl_3$.

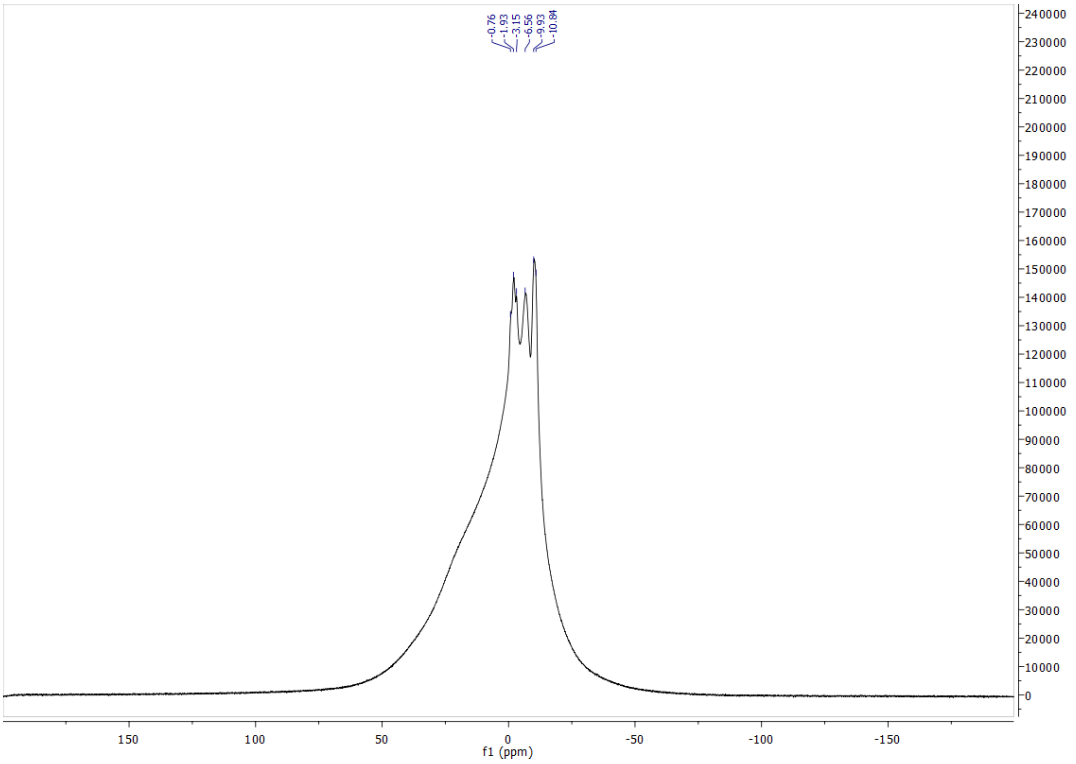

Figure S4. ^{11}B of **2** in $CDCl_3$.

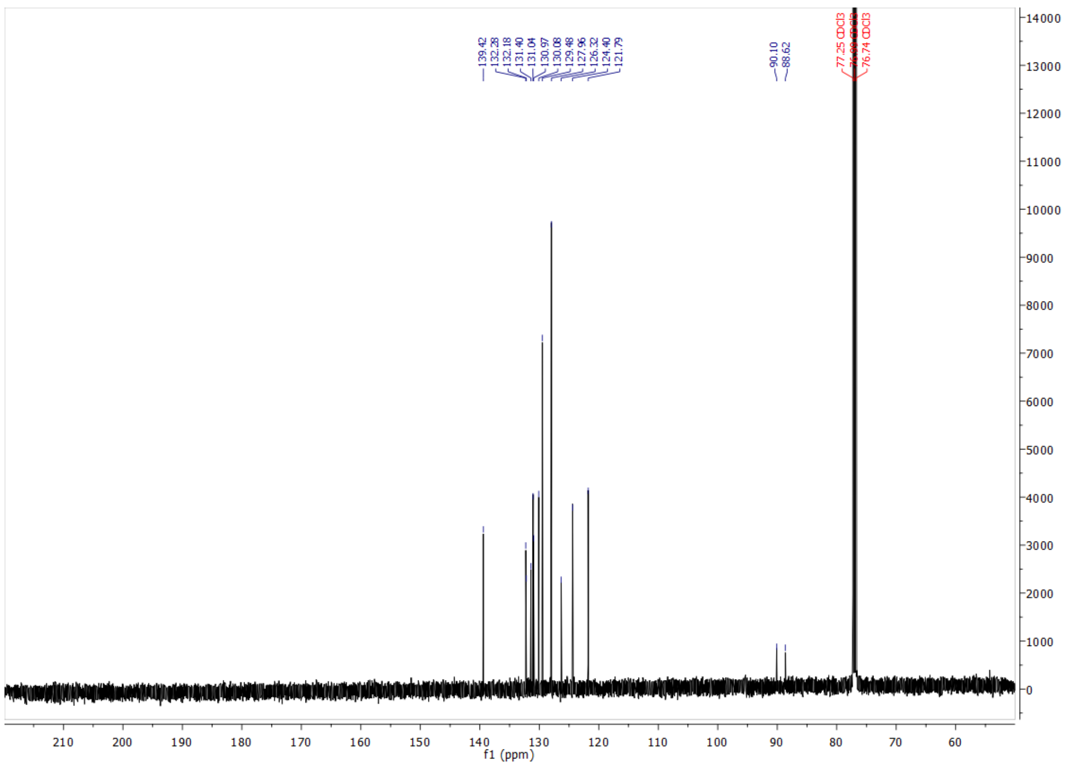

Figure S5. ^{13}C of **2** in $CDCl_3$.

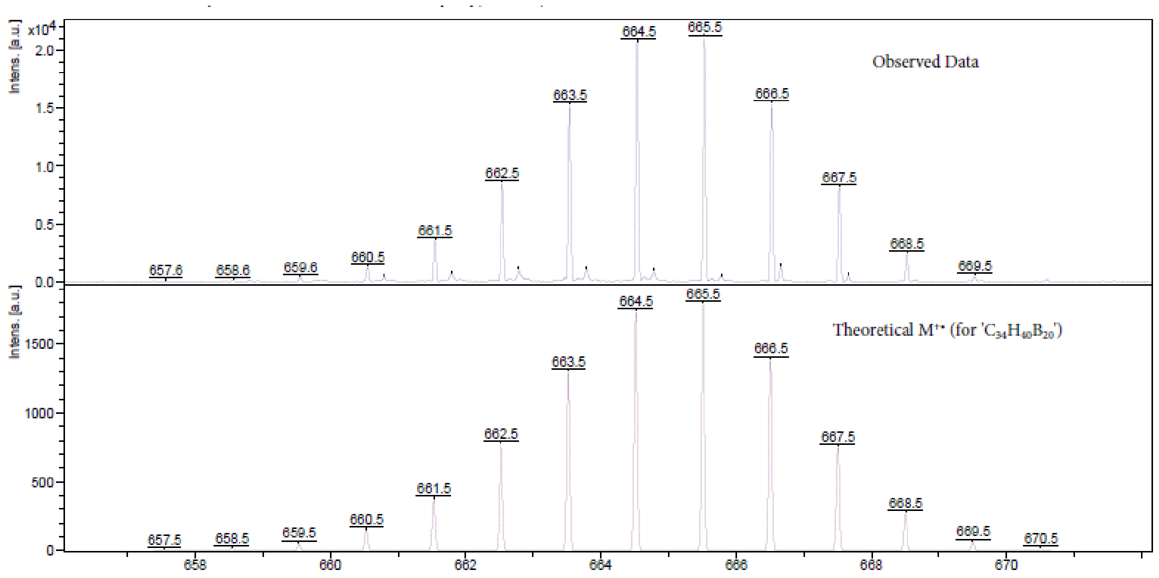

Figure S6. High-resolution MALDI-ToF mass spectrum of **2**. The expected ions were observed at m/z 665 at high resolution. For accurate mass measurement the monoisotopic peak is measured (m/z 657). As Boron-10 exists naturally at only 20% abundance with 20 Boron-10 atoms it is not practicable to perform accurate mass measurement on this compound.

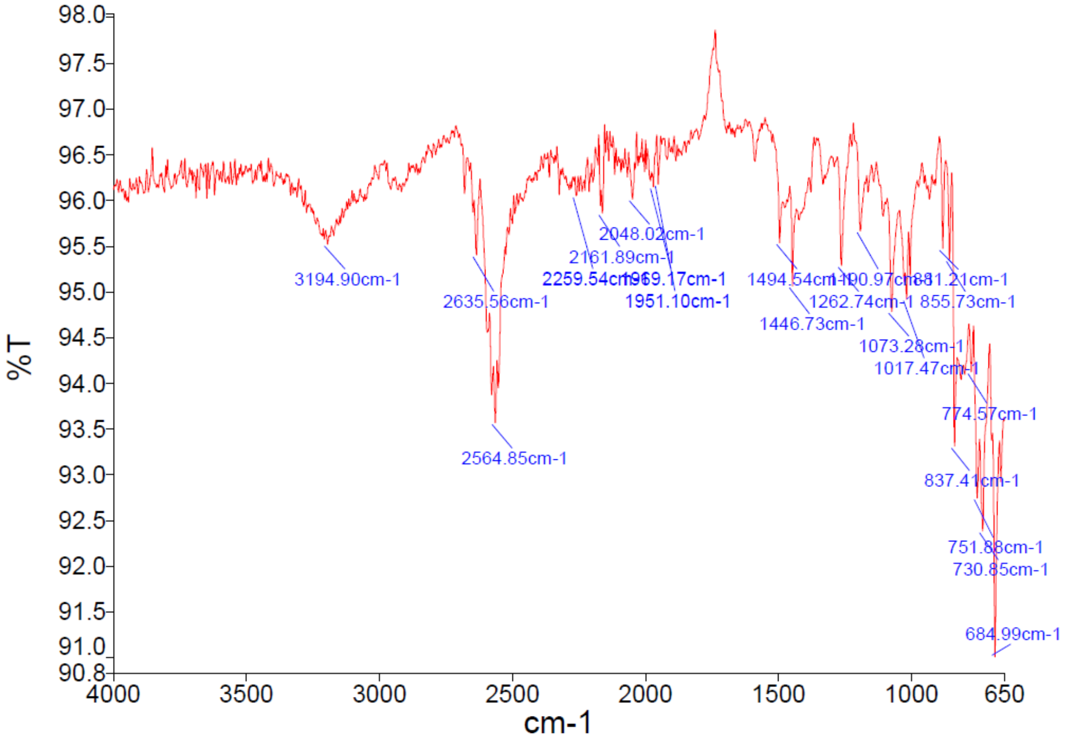

Figure S7. Infrared spectrum of **2**.

The X-ray crystal structure of 1

Crystal data for **1**: $C_{34}H_{20}$, M = 428.50, monoclinic, P21/c (no. 14), a = 4.04196(17), b = 24.5247(9), c = 11.0878(4) Å, β = 100.033(4)°, V = 1082.30(7) Å3, Z = 2 [Ci symmetry], Dc = 1.315 g cm^{-3}, μ(Cu-Kα) = 0.567 mm^{-1}, T = 173 K, yellow plates, Agilent Xcalibur PX Ultra A diffractometer; 2096 independent measured reflections (Rint = 0.0306), F2 refinement,[5,6] R1(obs) = 0.0455, wR2(all) = 0.1259, 1625 independent observed absorption-corrected reflections [|Fo| > 4σ(|Fo|), 2θmax = 147°], 154 parameters. CCDC 1836780. The structure of **1** sits across a centre of symmetry at the middle of the C8–C8A bond.

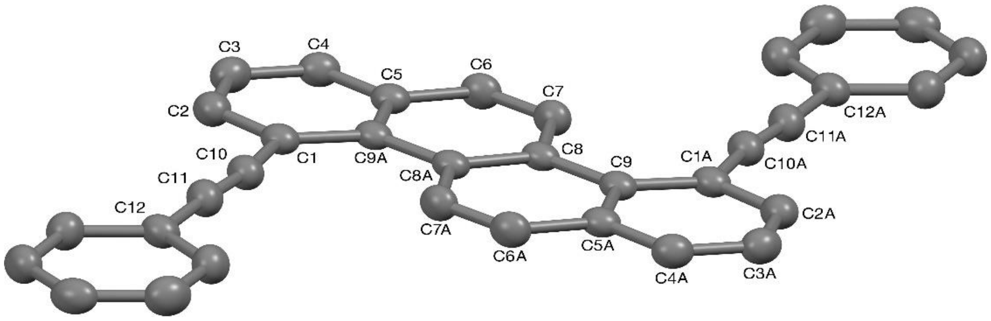

Figure S8. The crystal structure of **1** (50% probability ellipsoids). Protons omitted for clarity.

The X-ray crystal structure of 2

Crystal data for **2**: $C_{34}H_{40}B_{20}$, M = 664.86, orthorhombic, Pbca (no. 61), a = 15.6347(2), b = 14.20177(17), c = 32.3610(4) Å, V = 7185.43(16) Å3, Z = 8, Dc = 1.229 g cm^{-3}, μ(Cu-Kα) = 0.436 mm^{-1}, T = 173 K, yellow blocks, Agilent Xcalibur PX Ultra A diffractometer; 6970 independent measured reflections (Rint = 0.0349), F2 refinement,[5,6] R1(obs) = 0.0443, wR2(all) = 0.1200, 5518 independent observed absorption-corrected reflections [|Fo| > 4σ(|Fo|), 2θmax = 148°], 487 parameters. CCDC 1836781.

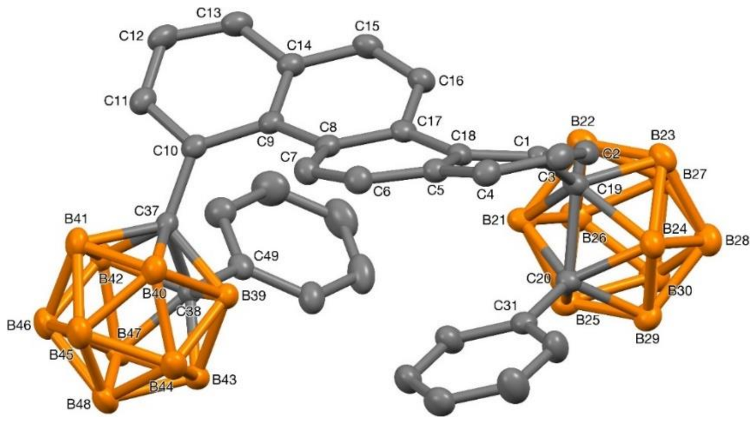

Figure S9. The crystal structure of **2** (50% probability ellipsoids). Protons omitted for clarity.

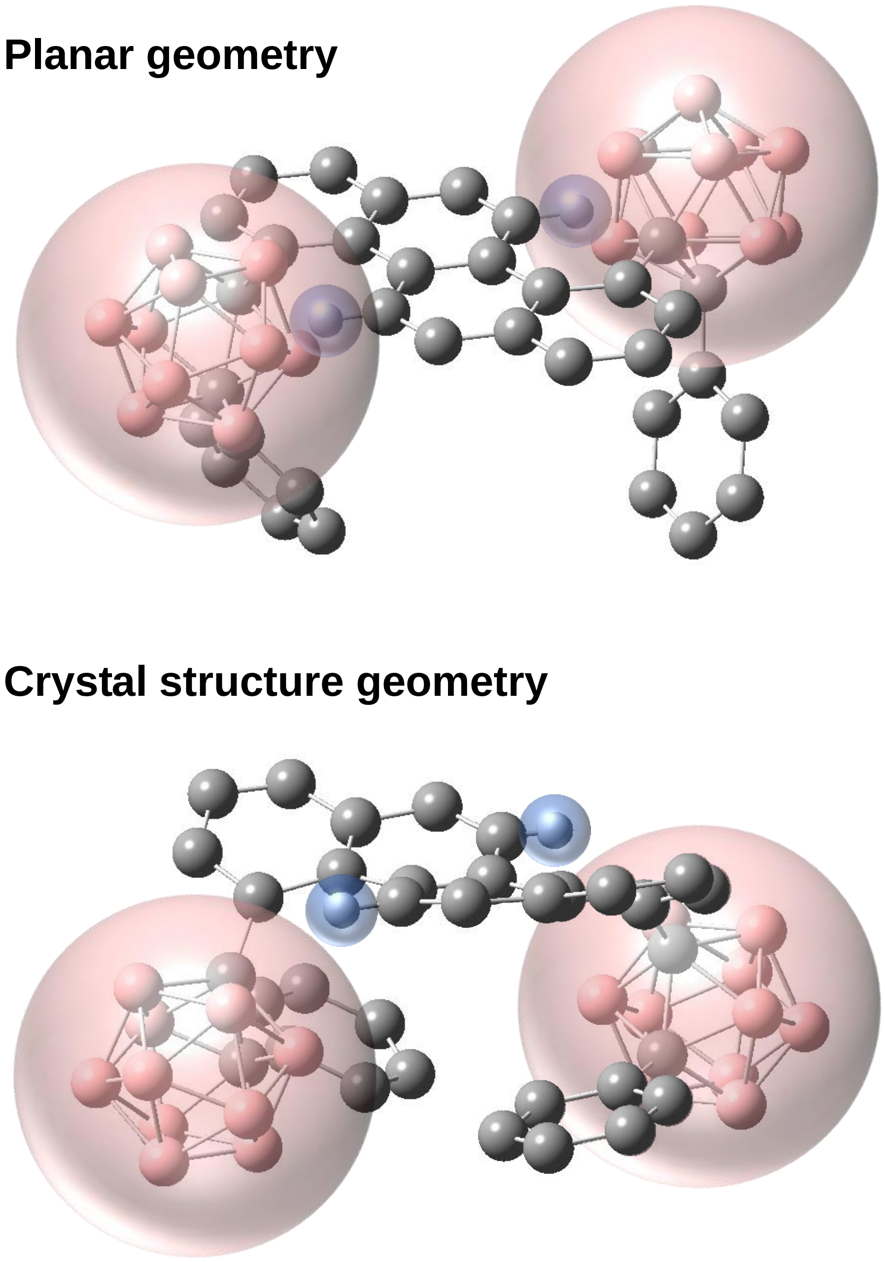

Figure S10. Approximate van der Waals (VDW) radius of carboranes (pink, shaded) and proximal aromatic protons (blue, shaded) of **2** in a theorized planar geometry (top) and measured crystal structure geometry (bottom). In the planar geometry the proximal protons are completely enveloped inside the VDW radius of the carborane, restricting all carborane rotation. In the crystal structure geometry, the deformation of the chrysene core enables the proximal protons VDW radius to separate completely from that of the carborane, allowing for limited rotation.

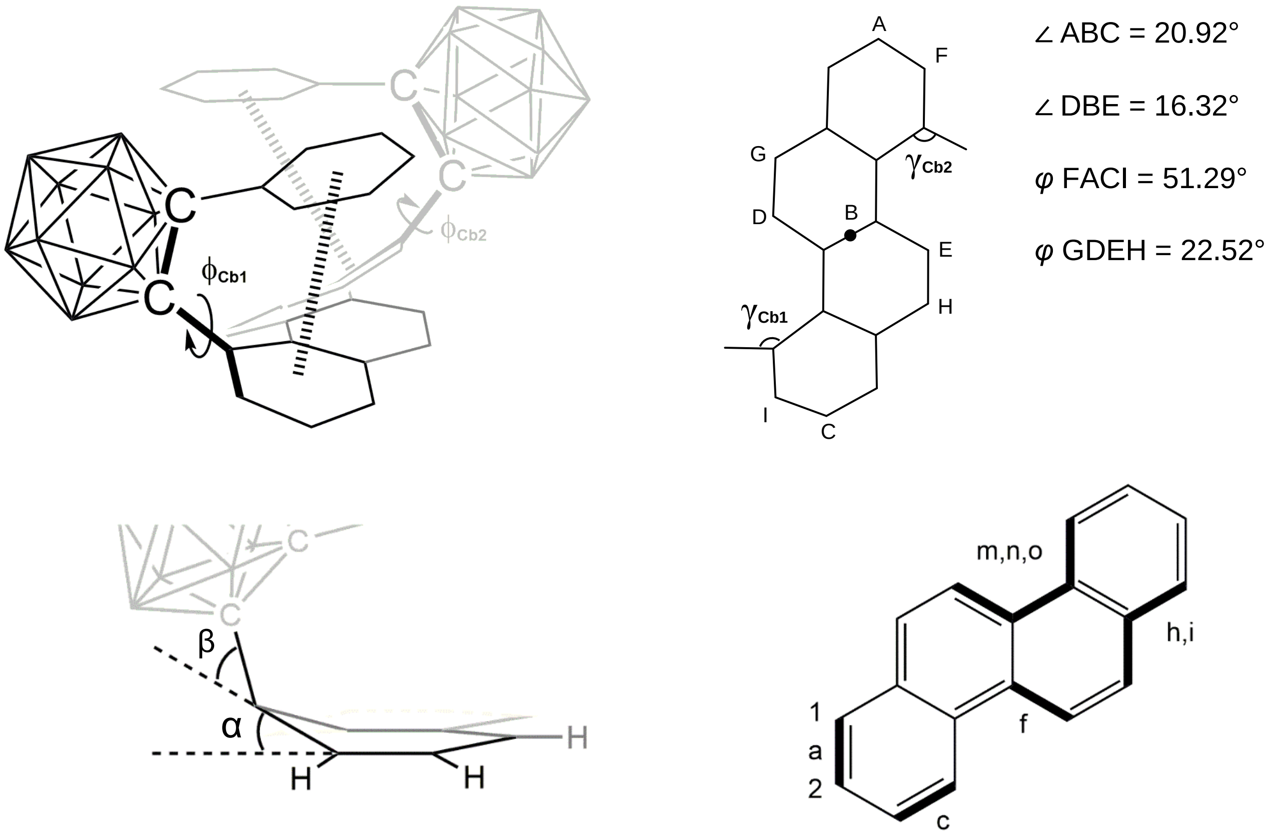

Carborane	α / °	β / °	γ / °	ϕ / °	π-π distance / Å	π-π ring plane angle / °
1	15.52	19.49	126.08	71.11	3.92	26.91
2	17.50	13.48	124.85	71.69	3.78	23.87

Figure S11. Specification of new quantities γ, ϕ and π-π stacking distances and angles, measuring the π-interactions between the carboranyl-phenyl ring and the nearest chrysenyl ring, as shown in the diagram. Angles α and β defined as in [5].

Table S12. Deformation angles α and β of **2** in comparison to similarly deformed carborane-containing acenes.

Compound	α / °	β / °	α + β / °
**2**$_{Cb1}$	**15.5**	**19.5**	**35.0**
**2**$_{Cb2}$	**17.5**	**13.5**	**31.0**
9,10-Bis(2-phenyl-1,2-dicarbadodecaboran-1-yl)anthracene[6]	21.5	6.4	27.9
5,12-Bis(2-phenyl-1,2-dicarbadodecaboran-1-yl)tetracene[7]	20.2	7.2	27.4
9-(2-phenyl-1,2-dicarbadodecaboran-1-yl)anthracene[8]	15.7	8.0	23.7
9-(1,2-dicarbadodecaboran-1-yl)anthracene[8,9]	13.6	7.2	20.8
9-(2-trimethylsilyl-1,2-dicarbadodecaboran-1-yl)anthracene[8]	13.5	4.5	18.0
9-(2-methyl-1,2-dicarbadodecaboran-1-yl)anthracene[8]	13.4	4.5	17.9

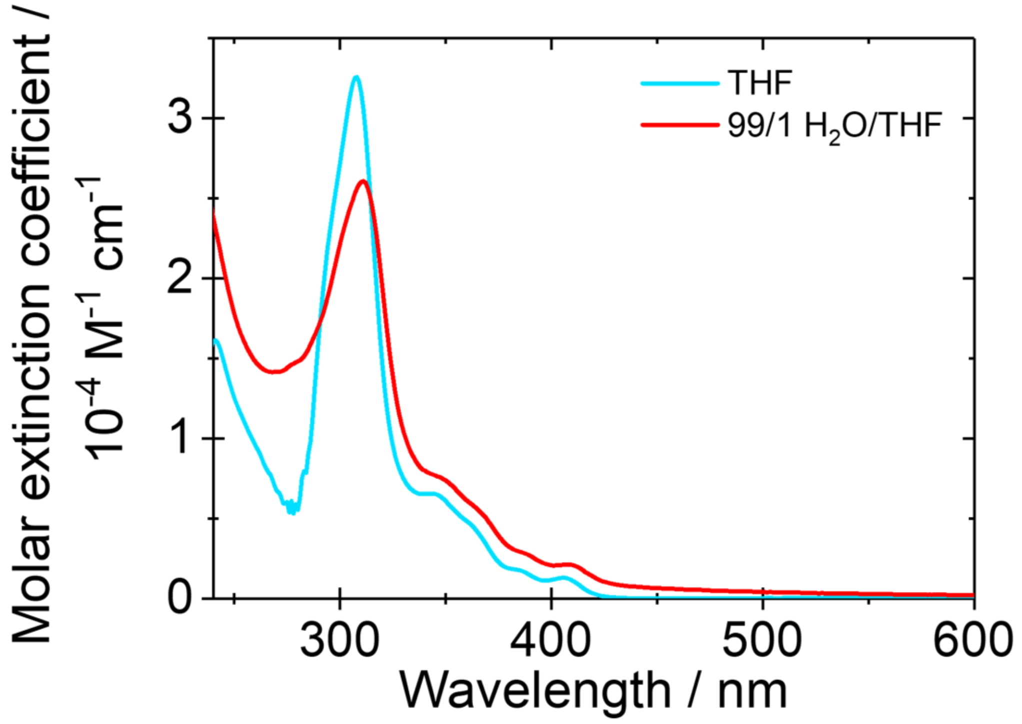

Figure S13. UV-Vis absorption of **2** in THF and H_2O:THF (v/v 99/1).

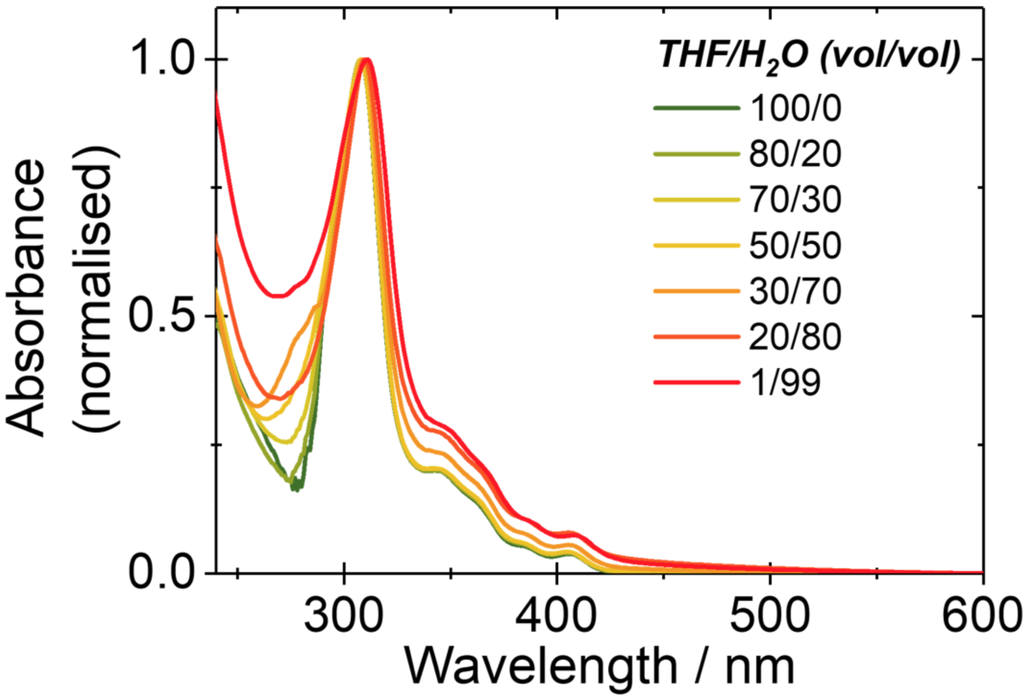

Figure S14. UV-Vis absorption of **2** in various H_2O:THF solvent compositions, normalized to ~300 nm peak. Concentrations ~1 x 10^{-4} M.

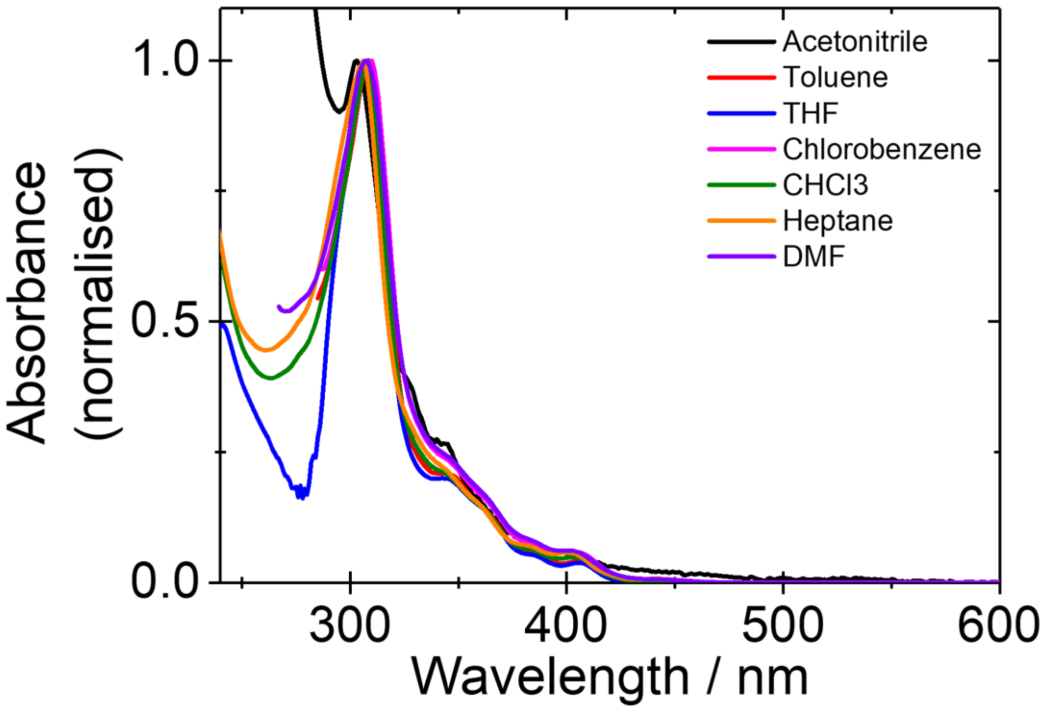

Figure S15. UV-Vis absorption of **2** in various solvents, normalized to ~300 nm peak. Concentrations ~1 x 10^{-4} M.

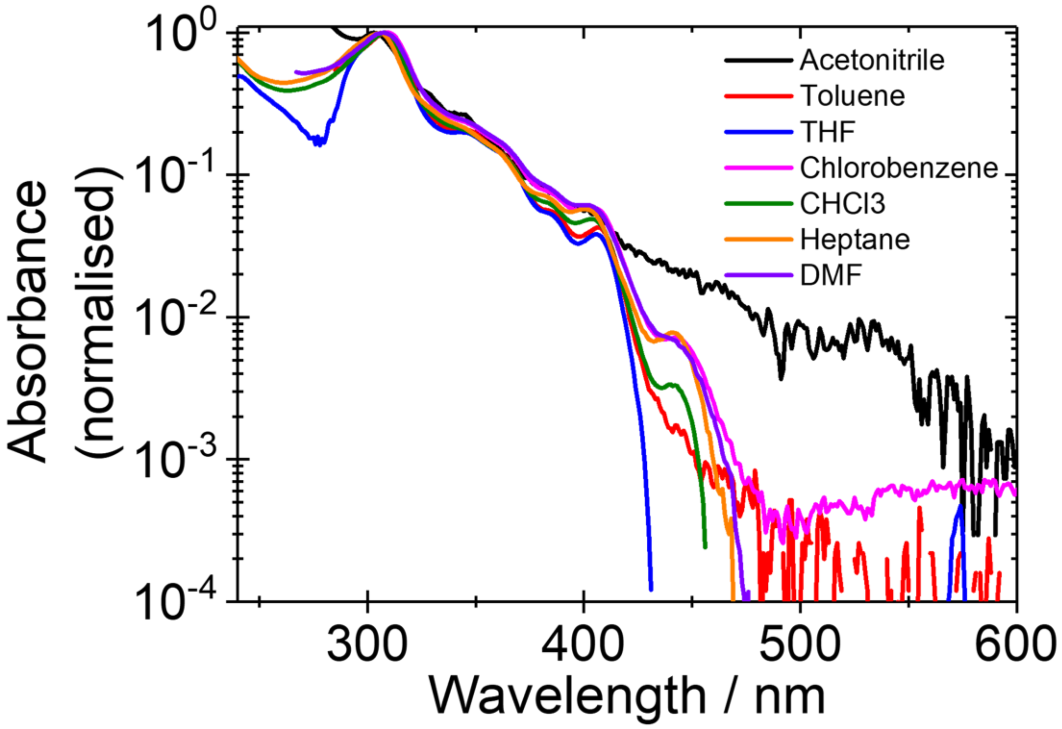

Figure S*16*. UV-Vis absorption of **2** in various solvents, with absorbance normalized to ~300 nm peak and presented on a log scale. Concentrations ~1 x 10^{-4} M.

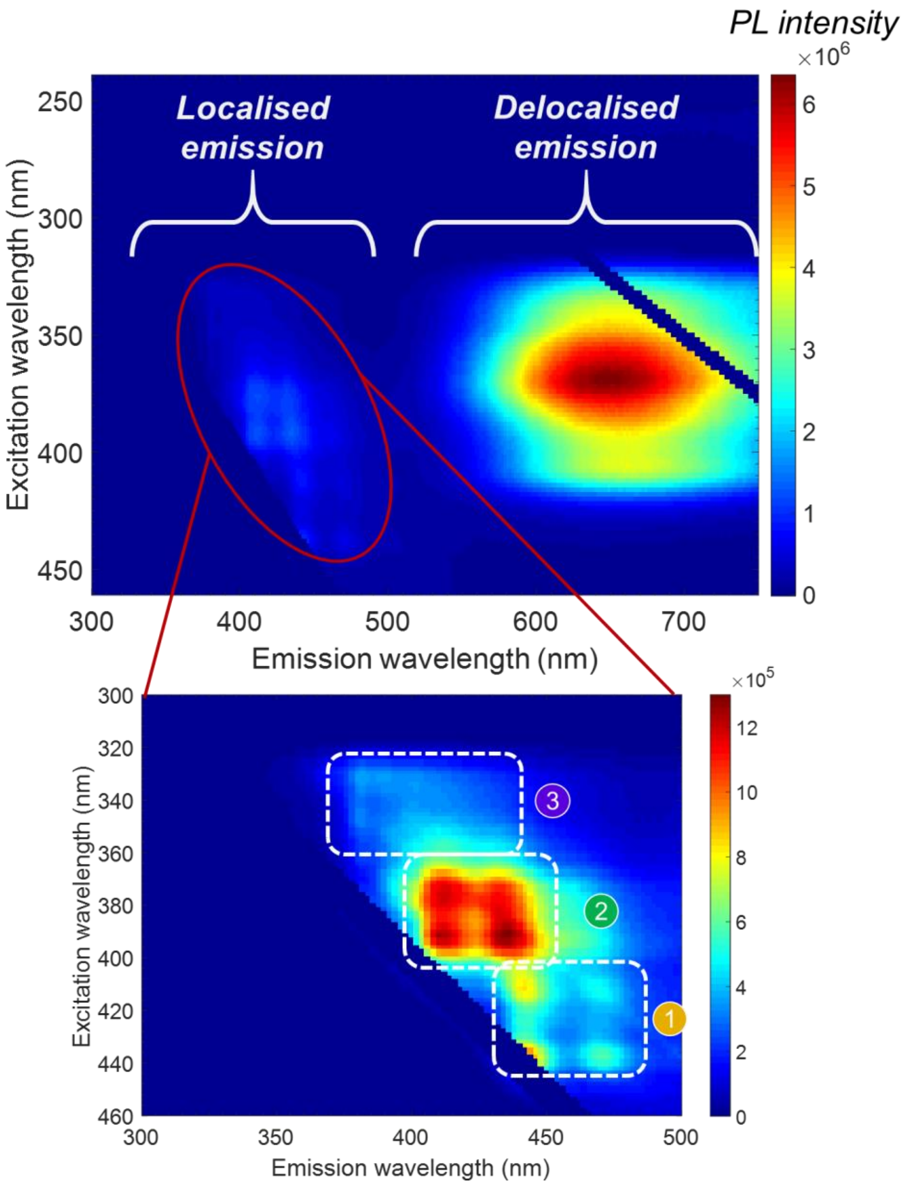

Figure S17. 2D photoluminescence emission map of **2** in THF (1 x 10^{-4} M). Data removed at one and two-times the excitation wavelength. We identify at least three sets of correlated absorption and emission peaks, with 0-0 PL vibronic emission peaks centred at 380, 410, 440 nm.

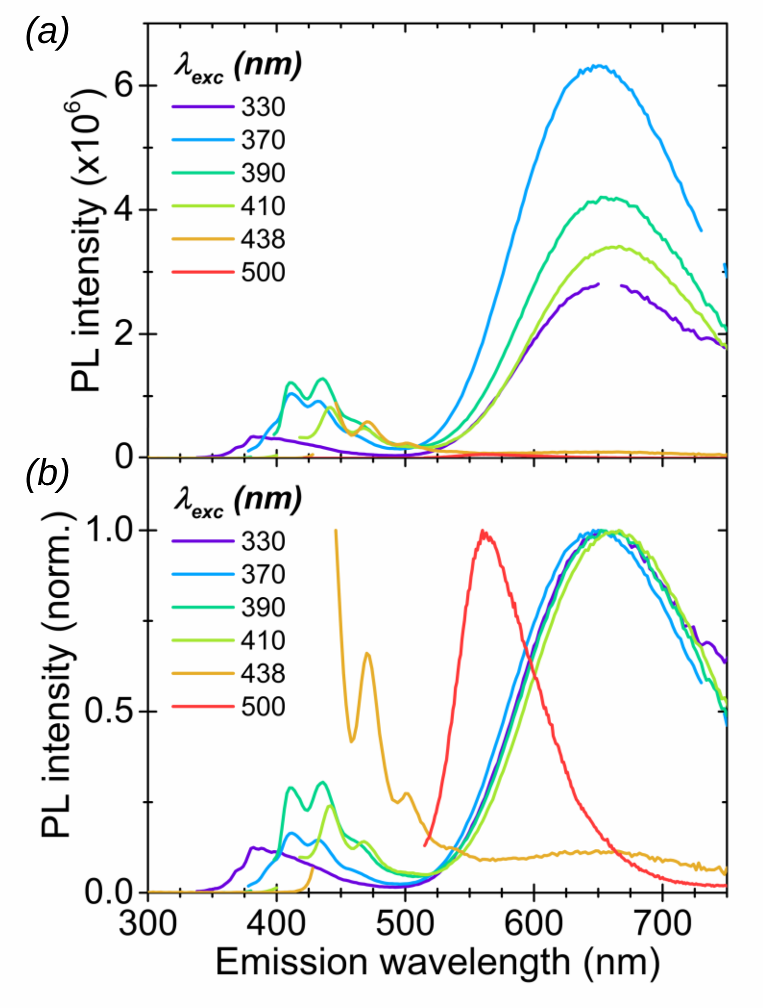

Figure S18. Photoluminescence emission of **2** in THF (1 x 10^{-4} M) at various excitation wavelengths (a), and normalised (b).

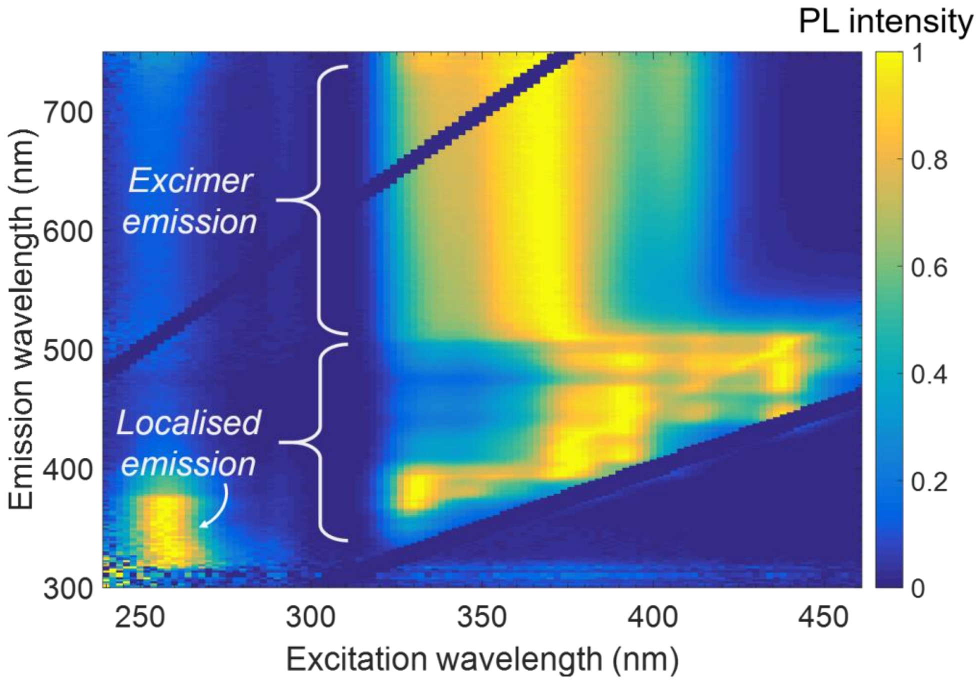

Figure S19. 2D photoluminescence excitation map of **2** in THF (1 x10^{-4} M), normalised to the maximum PL intensity at each emission wavelength (each slice along the y axis corresponds to a normalised PLE diagram). Data removed at one and two-times the excitation wavelength.

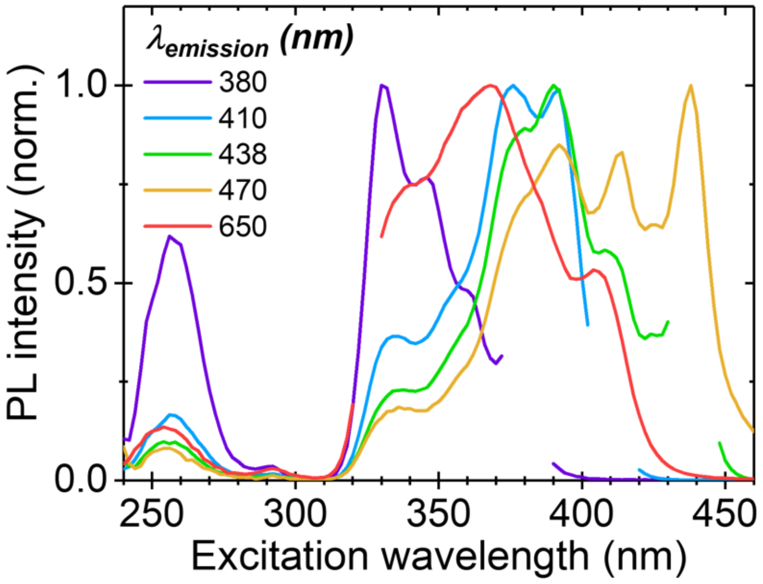

Figure S20. Photoluminescence excitation (PLE) of **2** in THF (1 x 10^{-4} M) at various emission wavelengths.

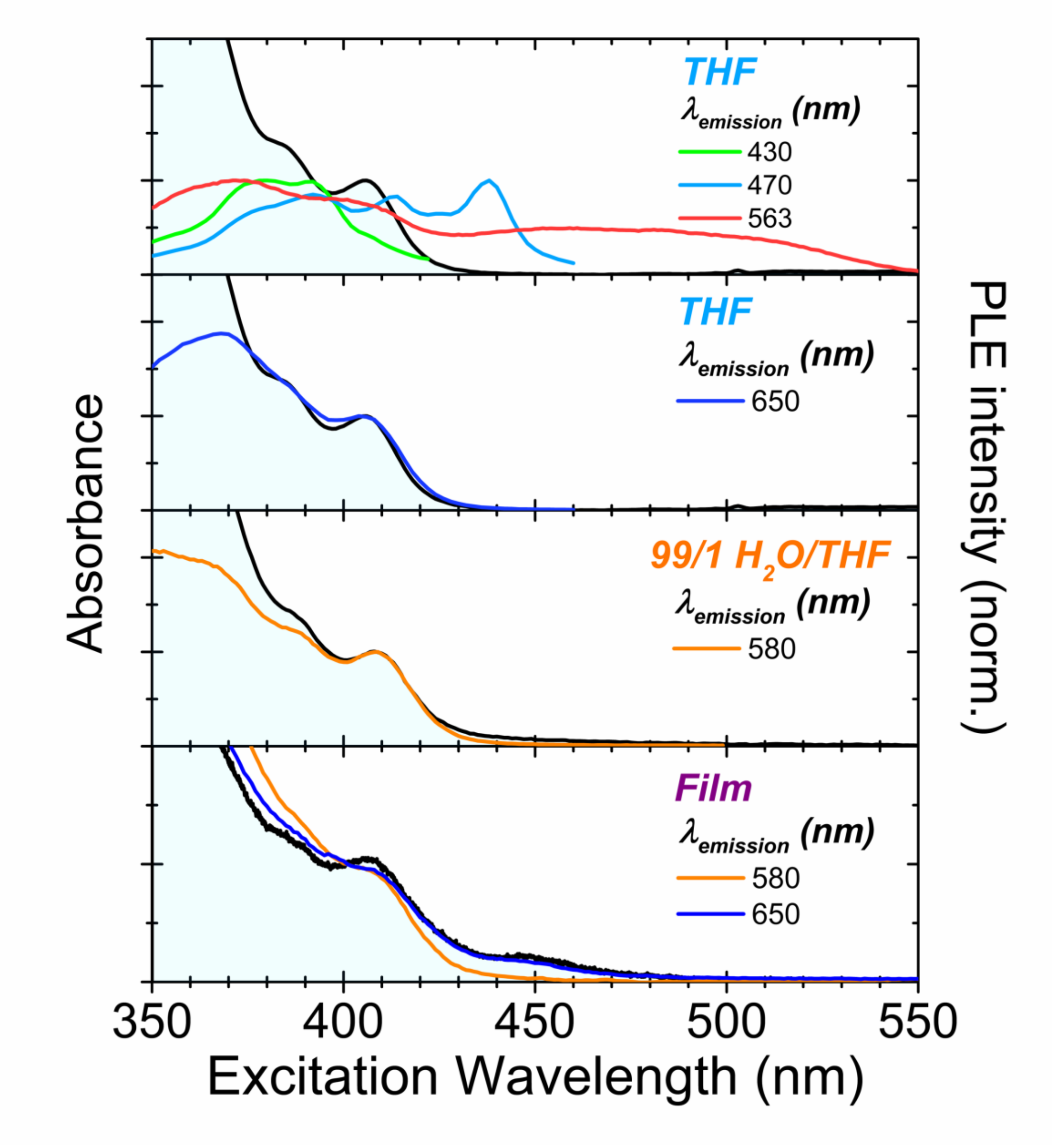

Figure S21. UV-Vis absorption (shaded blue) and selected PLE of **2** in THF, 99/1 H_2O/THF and solid-state film. Solution concentrations ca. 10^{-4} M.

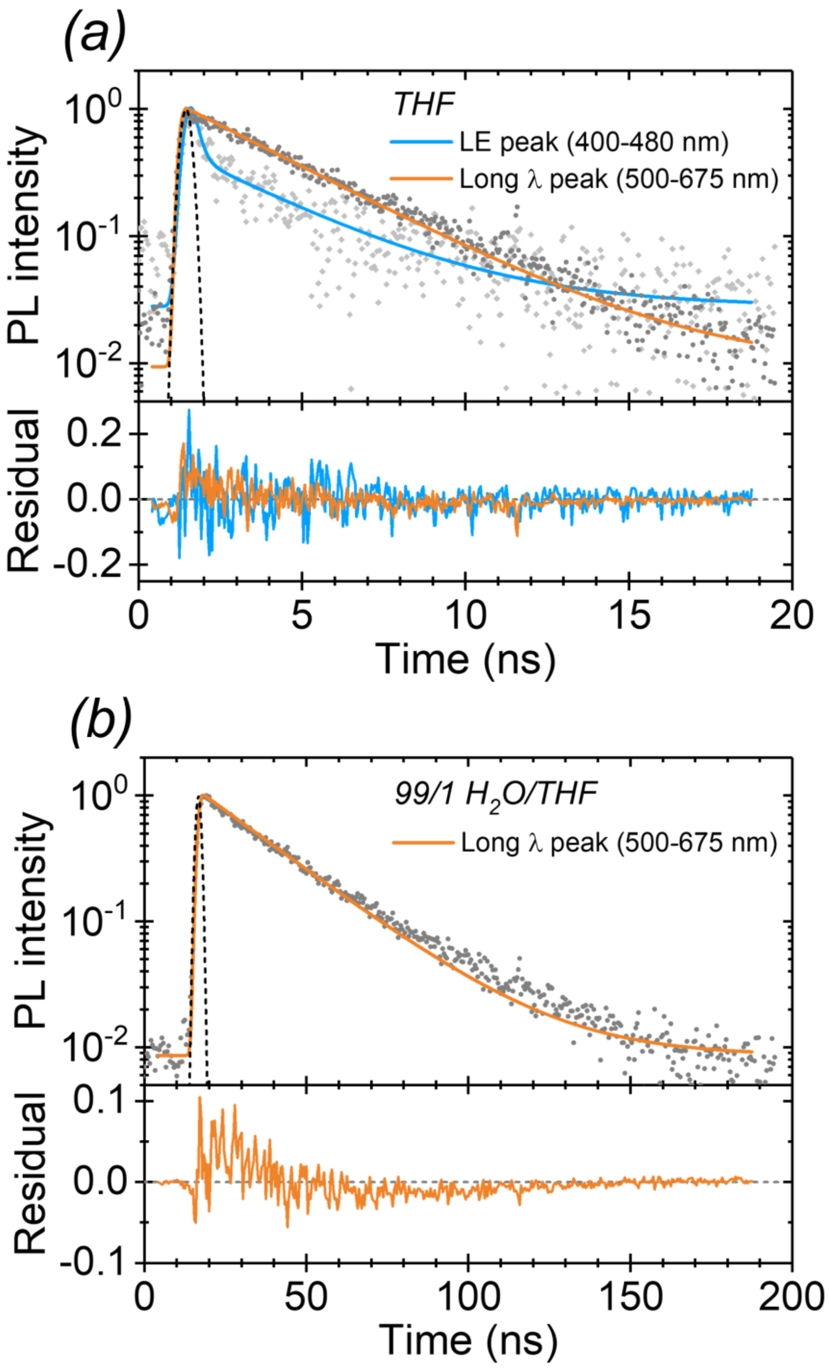

Figure S22. Time-resolved PL kinetics of localized (LE) and long wavelength emission regions for **2** in THF solution (1 x 10^{-4} M) (a), and in a H_2O/THF (v/v 99:1) solution (1 x 10^{-4} M) (b). The kinetics of the localized and long wavelength emissions are fitted with double and single exponential functions respectively, convolved with a gaussian peak (black dashed lines) representing the instrument response function. Lower panels show the residuals of the fits.

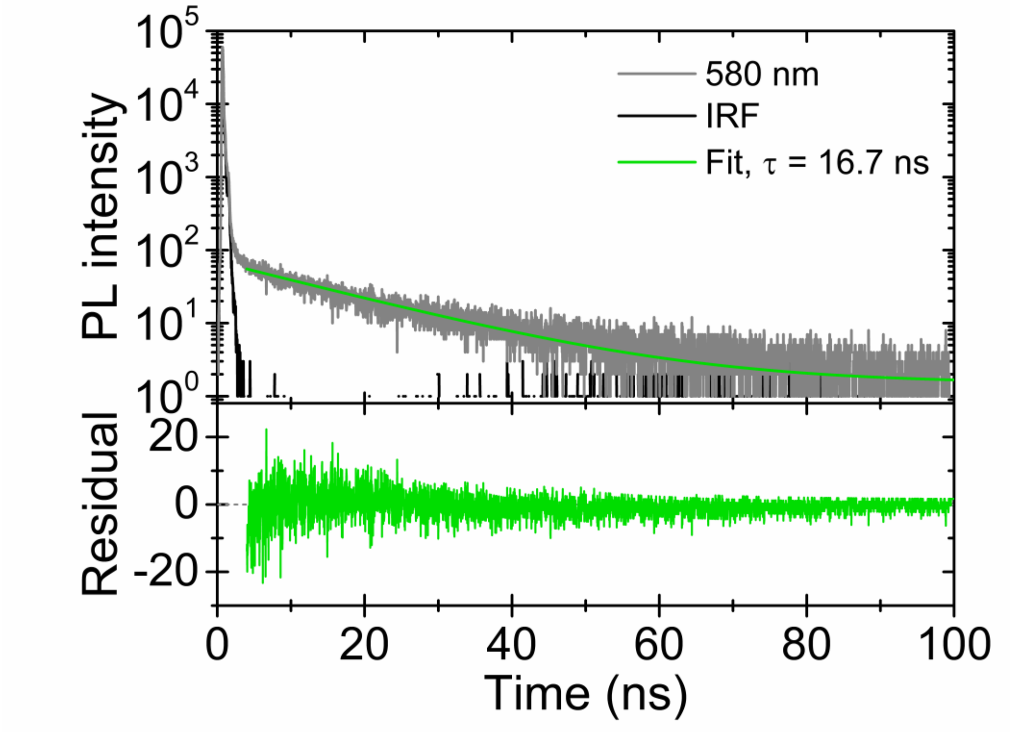

Figure S23. Time-resolved PL kinetics of the 580 m emission region for a thin film of **2** on glass. The kinetics of the emission is fitted with a single exponential function whilst the the instrument response function is also shown (black line). Lower panels show the residuals of the fit.

Table S24. Summary of the PL, PL quantum efficiency (PLQE) and time-resolved PL (TRPL) data.

Condition	λ_{em} / nm	Φ_{PL}	τ / ns
THF	436<br>657	3.1<br>3.1	0.18 ± 0.1<br>3.3 ± 0.1
H_2O:THF (v/v 99/1)	585	32	23 ± 1
Polycrystalline	583	55	17 ± 2[a]

[a]Measurement carried out on a film sample.

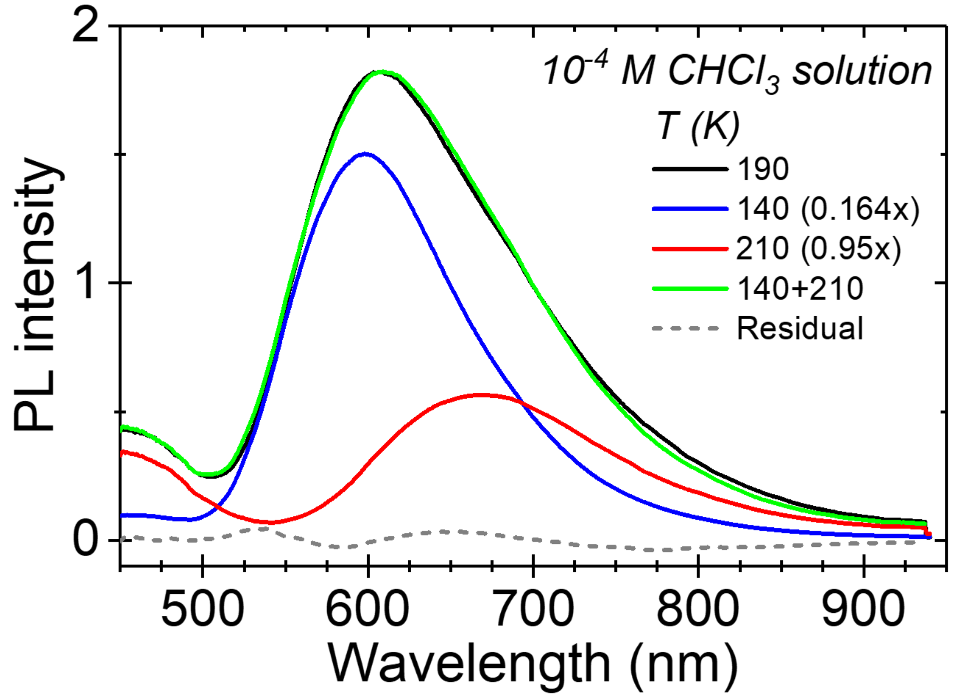

Figure S25. Temperature dependent photoluminescence emission of **2** in CHCl3, at 140, 190 and 210 K, with peak fitting of the 190 K emission as a function of linearly scaled 140 and 210 K emissions.

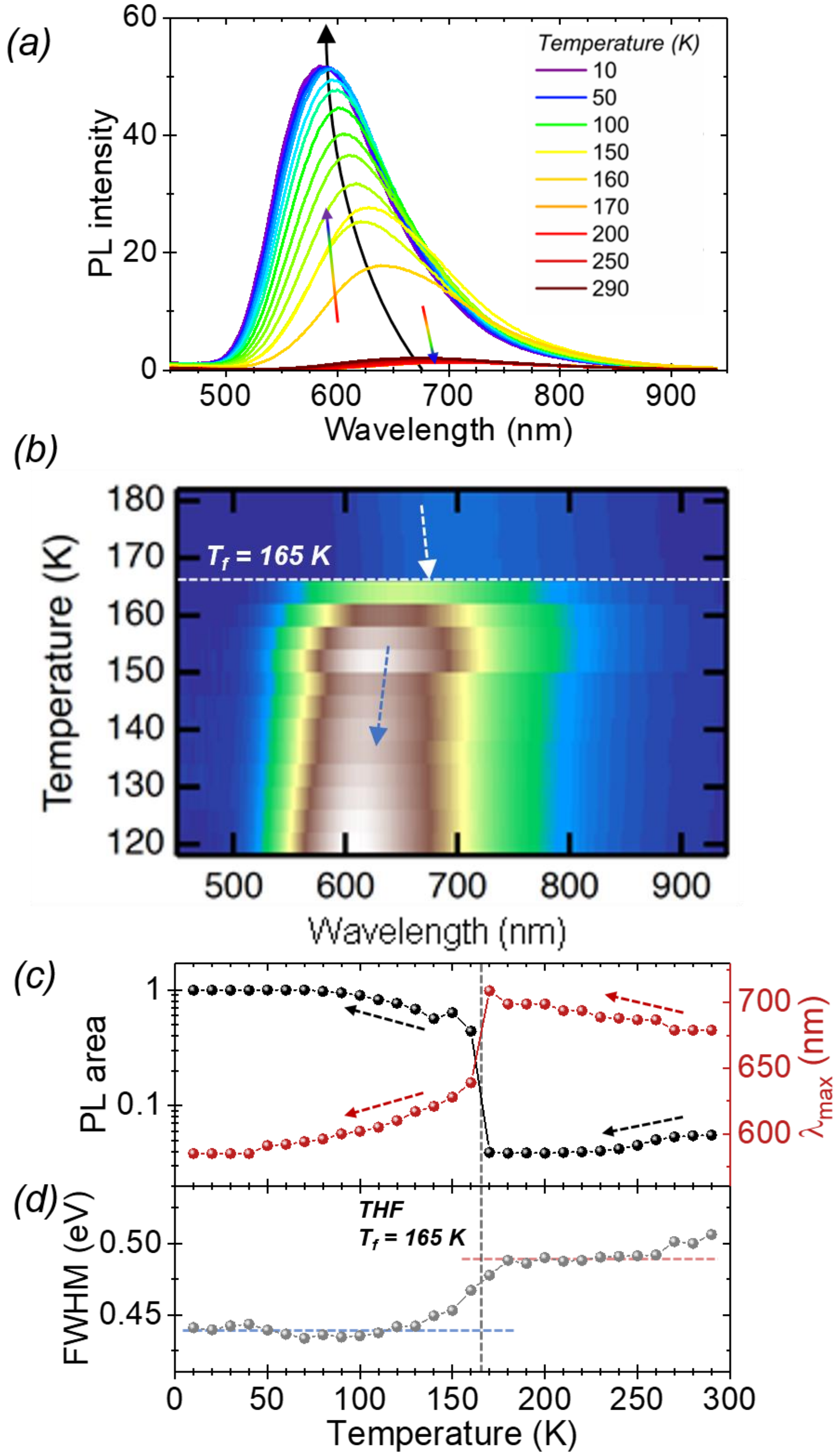

Figure S26. Photoluminescence emission (PL) of **2** (a) in THF (a); as a 2D contour map (b); PL area ratio and long wavelength emission λ_{max} (c); and full width half maximum (FWHM) (d), as a function of temperature. Concentration ca. 10^{-4} M.

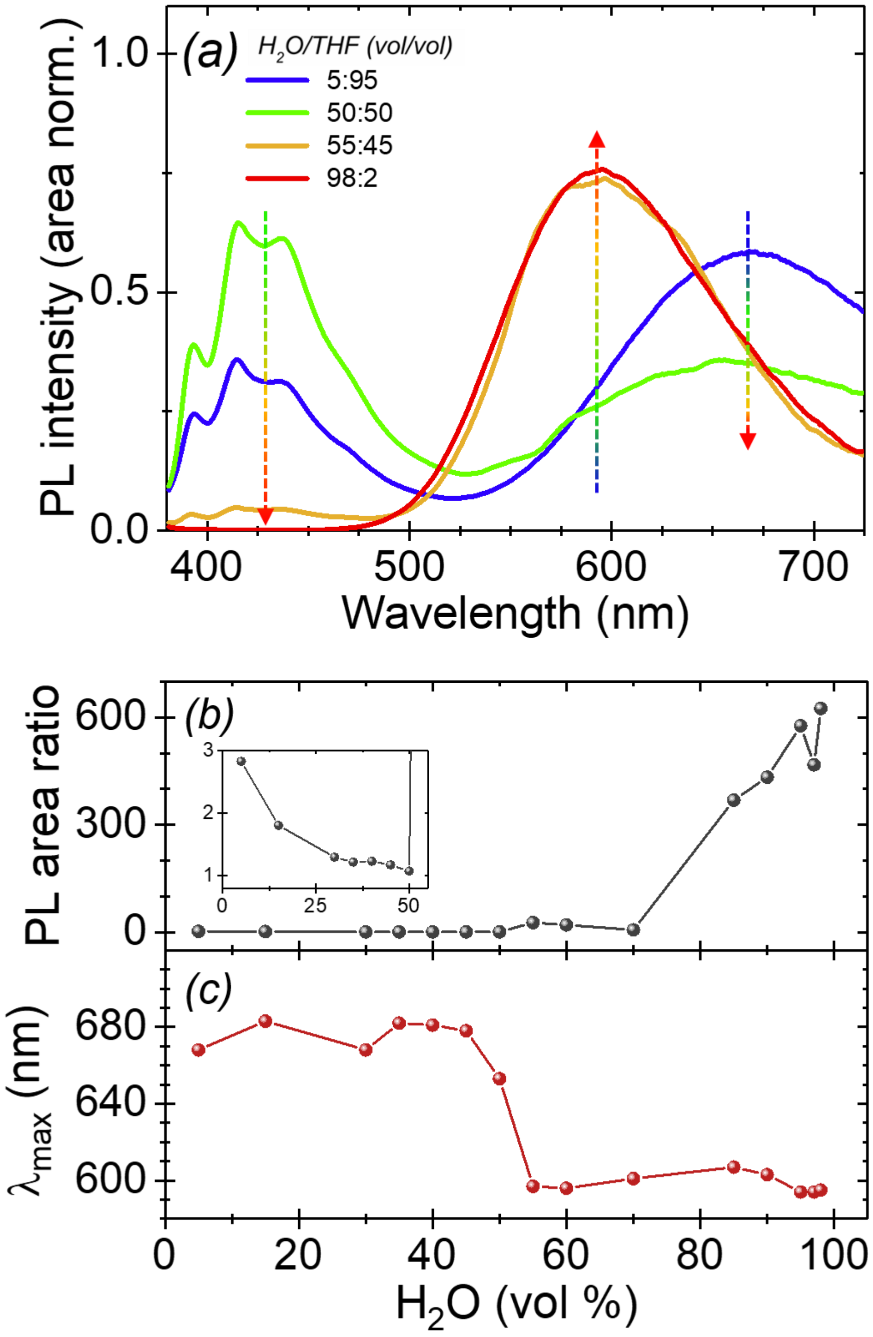

Figure S27. Photoluminescence emission of **2** at a range of varying H_2O/THF v/v (a), PL area ratio (b) and long-wavelength emission λ_{max} (c) as a function of H_2O vol%.

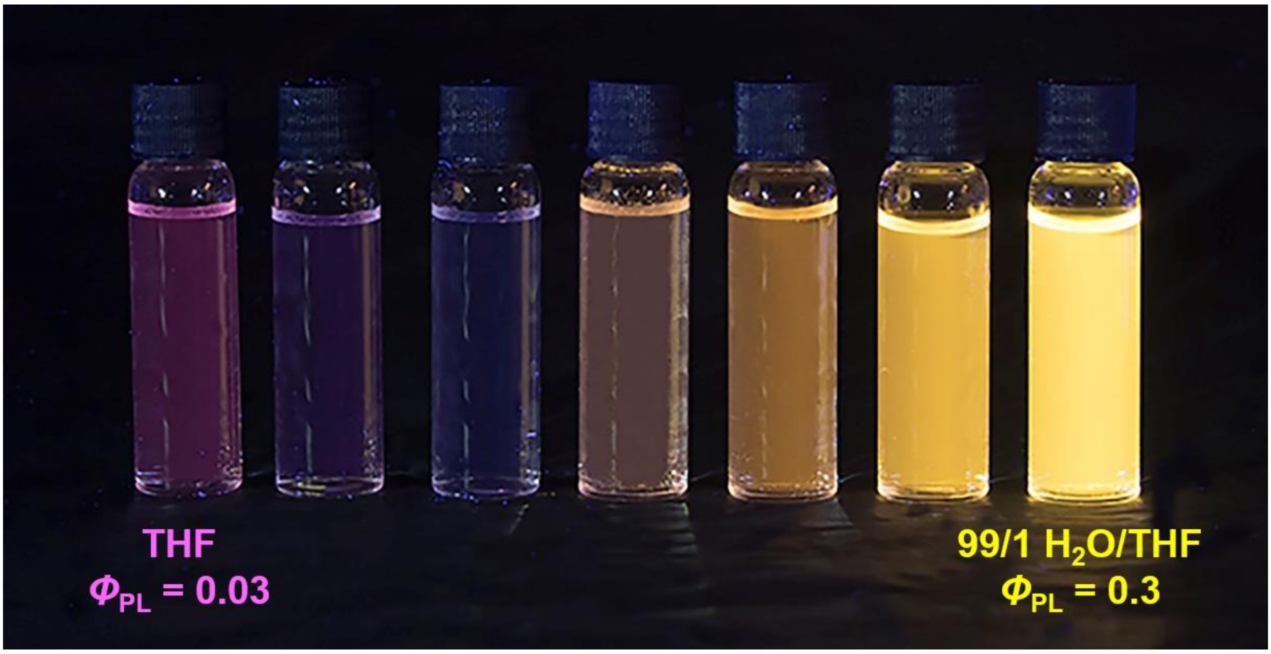

Figure S28. Solutions of **2** with varying H_2O:THF compositions, under UV excitation at 365 nm. From left to right, H_2O/THF v/v = 0/100, 25/75, 50/50, 80/20, 90/10, 95/5, 99/1.

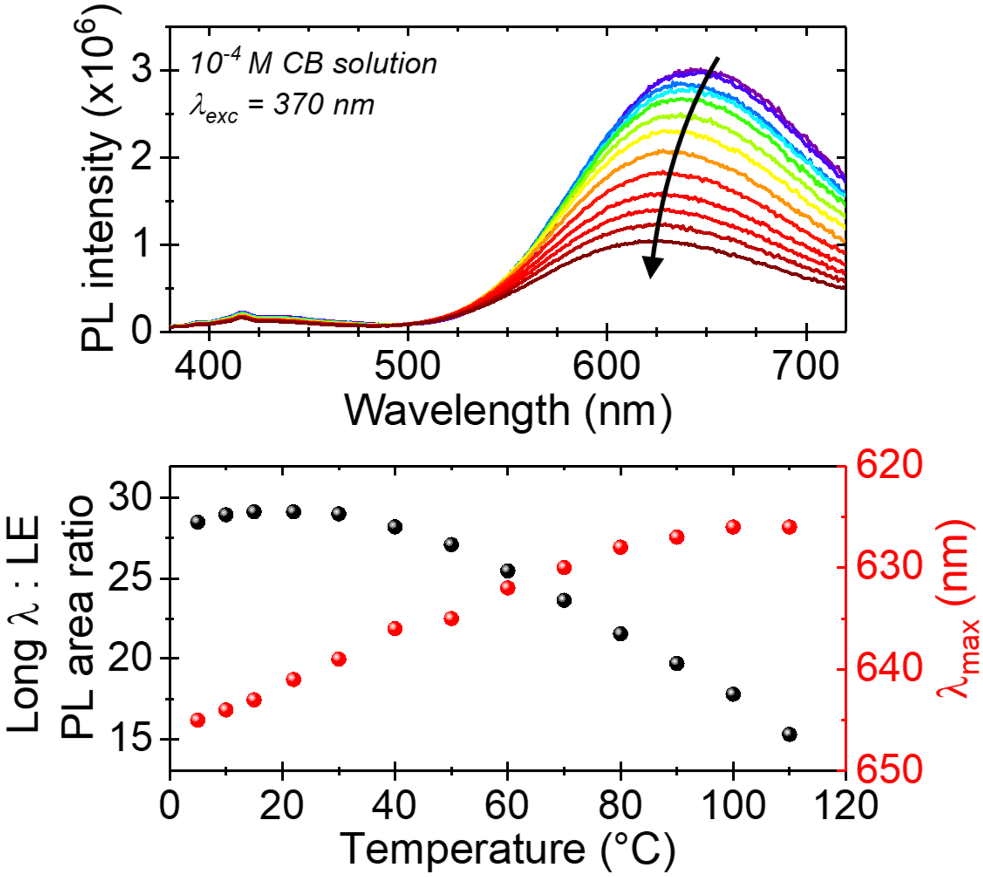

Figure S29. Photoluminescence emission of **2** in chlorobenzene (a), and long λ_{max} : LE area ratio and λ_{max} (b) as a function of termperature (> R.T.).

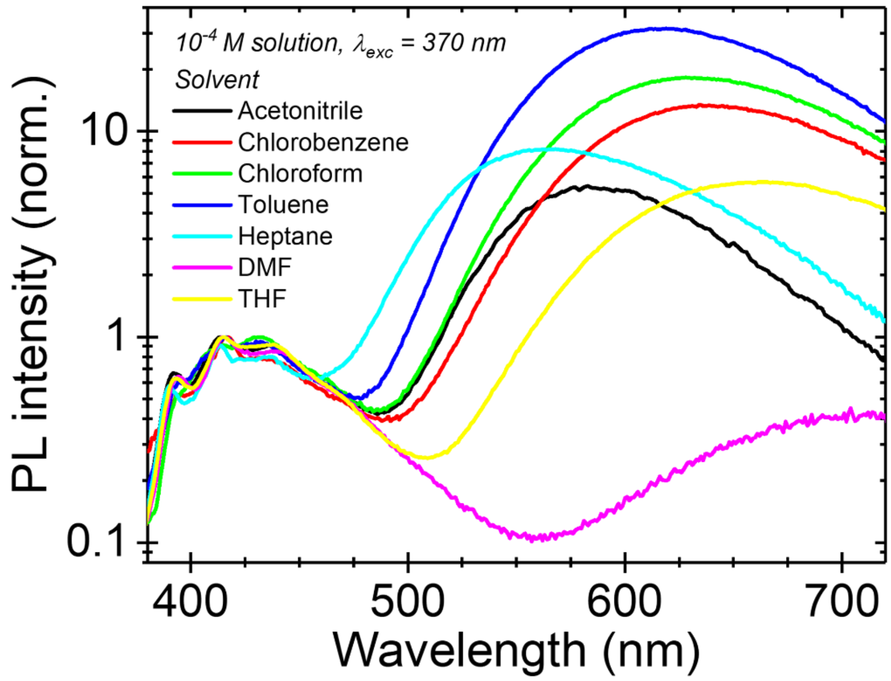

Figure S30. PL of **2** in selected solvents normalized to the vibronic region.

Table S31. Solvent quality parameters for those used in the solvatochromism study.

Solvent	Solvent dielectric constant (ε)	Refractive index (n)	Orientation polarisability (Δf)	Hildebrand polar parameter / $MPa^{1/2}$
Acetonitrile	37.5	1.3441	0.305416	18.0
Chlorobenzene	5.62	1.5248	0.142936	4.3
Chloroform	4.81	1.4459	0.148262	3.1
DMF	36.7	1.4305	0.274380	13.7
Heptane	2.1	1.3855	0.021506	0.0
THF	7.6	1.4050	0.210557	5.7
Toluene	2.38	1.4961	0.013474	1.4

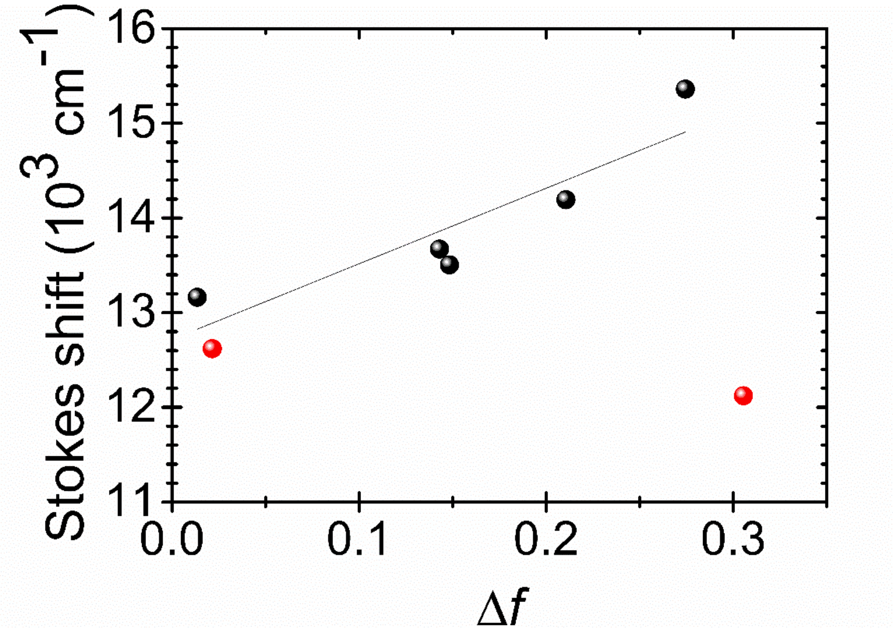

Figure S32. Lippert-Mataga plot[10] of the long wavelength peak of **2** with good solvents, as defined by Hildebrand solubility parameters of 9.0 ± 0.5 cal$^{1/2}$ cm$^{-3/2}$, in black and bad solvents in red. The trendline for good solvents shows a positive linear correlation.

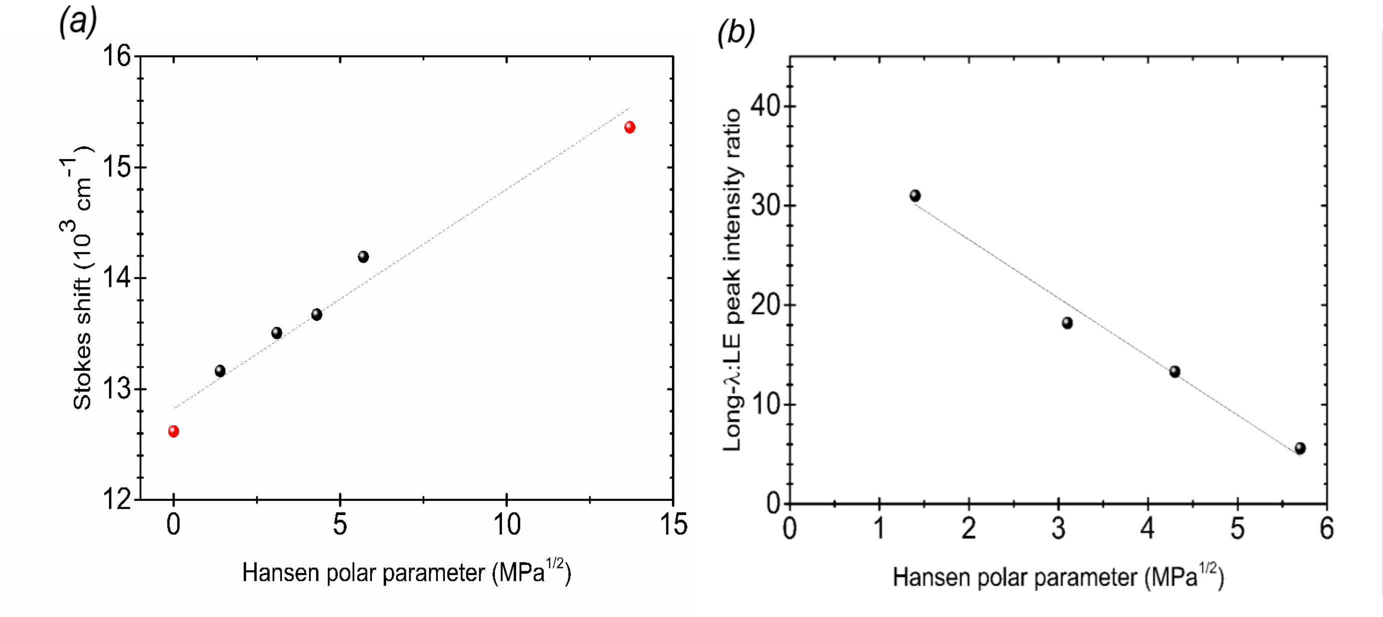

Figure S33. Stokes shift of **2** in selected solvents (a) and delocalized, long-λ:localized emission peak intensity ratio (b) as a function of solvent polarity, as defined by the Hansen solubility polar parameter.[11] Bad solvents are depicted in red. Stokes shift and PL peak intensities are plotted against the Hansen solubility polar parameter as an alternative means to test the effect of solvent polarity of the long-λ emission.

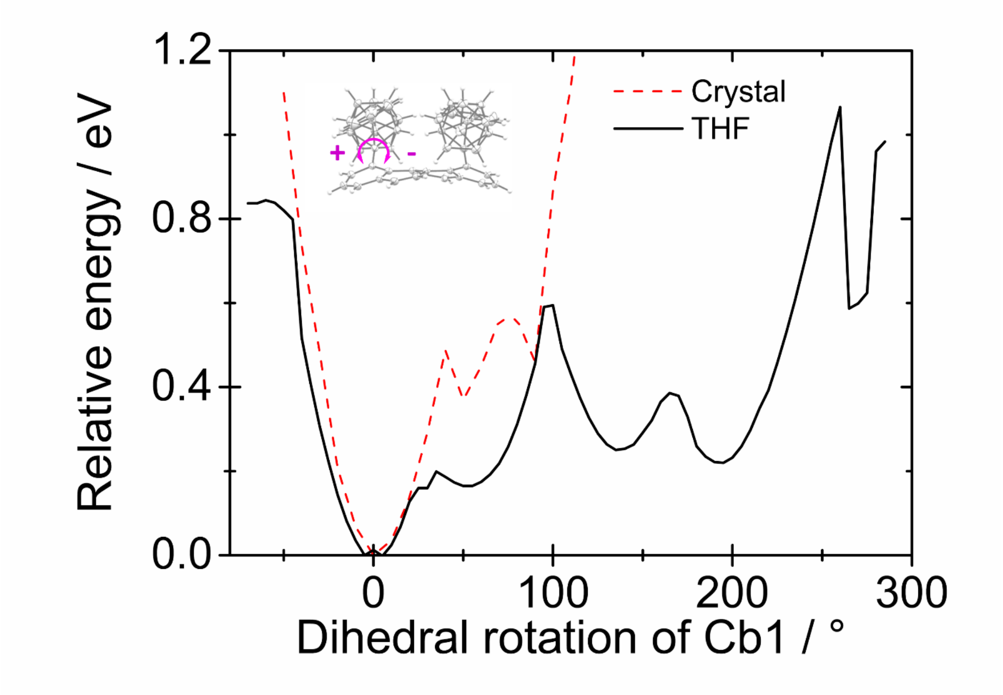

Figure S34. PES of Cb1 rotation of **2** in THF and in a crystal. THF PES revealed energy minima observed at 0°, 55°, 135°, 195°, where 0° is defined as the ground state dihedral angle (indicated) of Cb1. Crystal PES was calculated using a two-level ONIOM model, with DFT for the high-level, and molecular mechanics (MM) for the low-level, with electronic embedding turned-on.

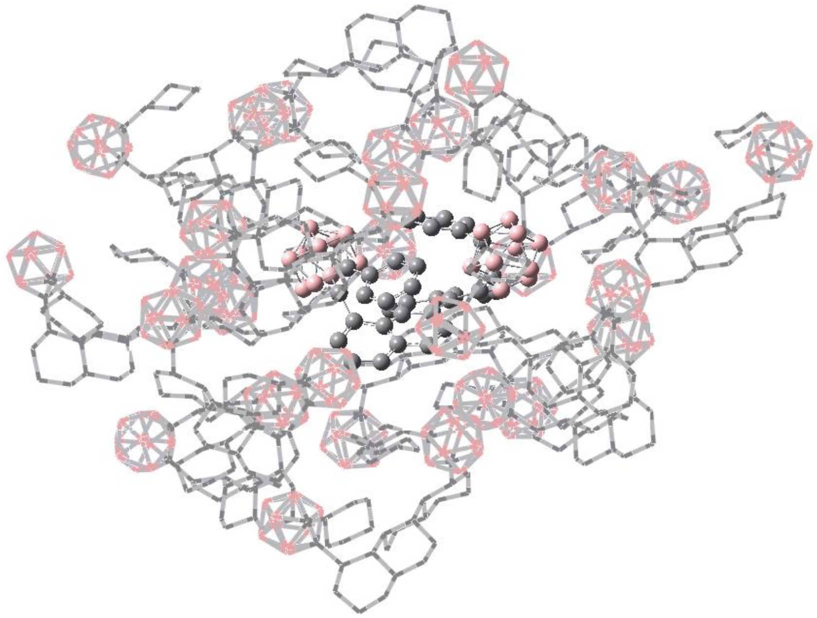

Figure S35. ONOIM model of **2** as used in crystal PES calculations. The central molecule is treated with high-level DFT (as above), and surrounding molecules are treated with molecular mechanics. Packing is as observed from single crystal x-ray analysis. Protons removed for clarity.

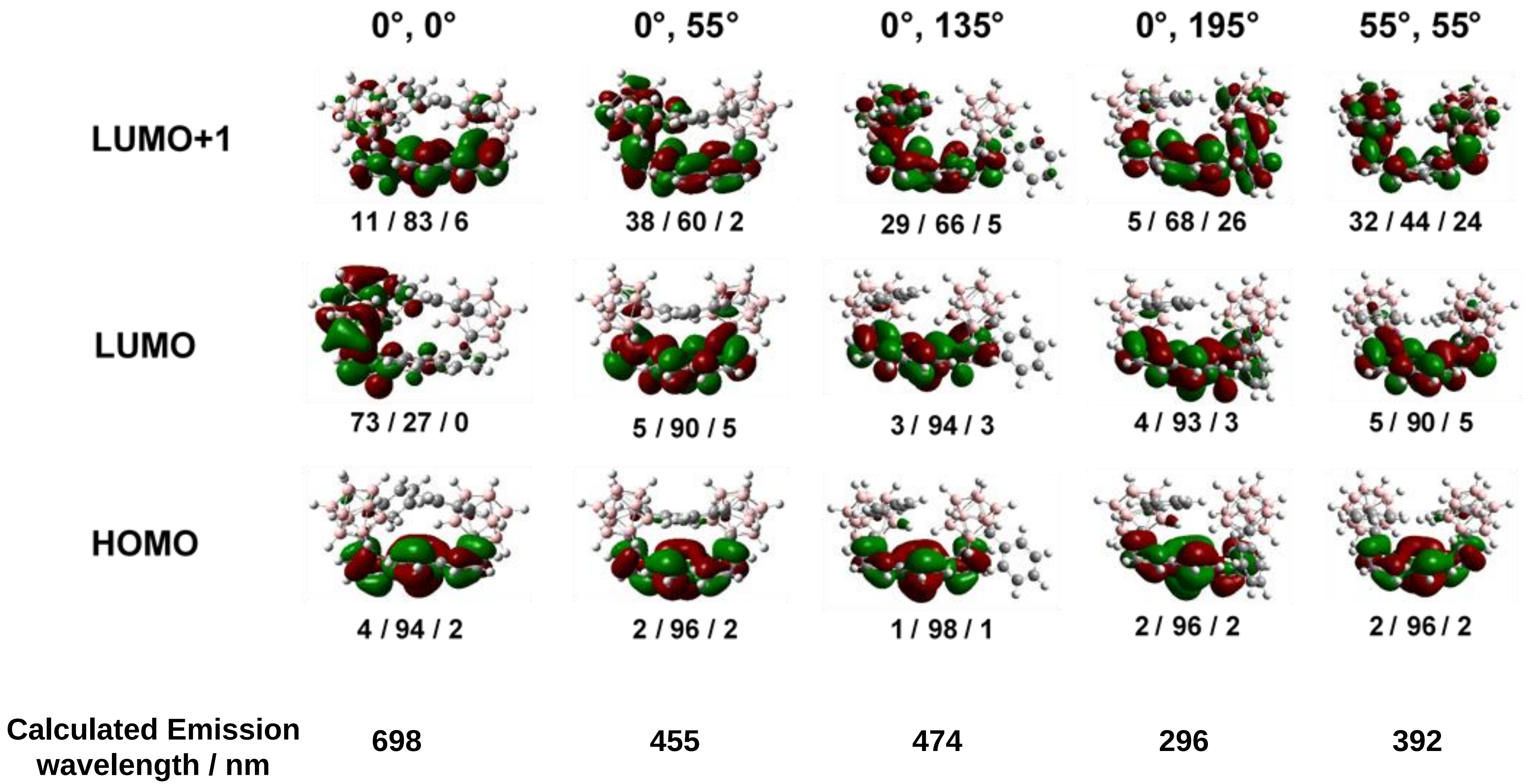

Figure S36. DFT calculated molecular orbital surfaces of HOMO, LUMO and LUMO+1 of the S1 excited state of **2** in THF. Calculated relative orbital contributions from Cb1 / chrysene / Cb2 are presented below each molecular orbital.[12] DFT calculated emission energies for the $S_1 \rightarrow S_0$ radiative relaxation in THF with non-equilibrium solvation are also shown.

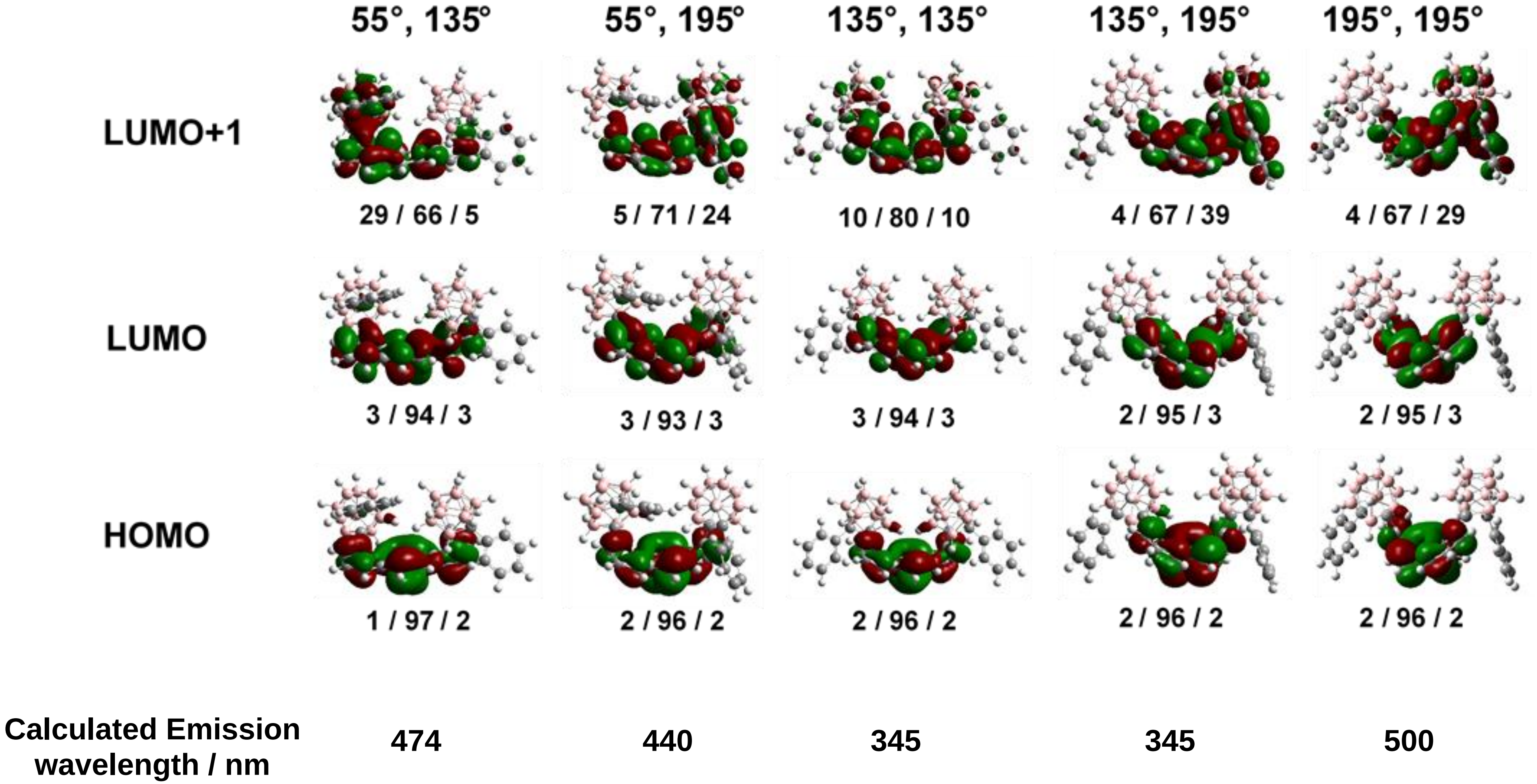

Figure S36. Continued.

E_{red}^{onset} vs Ag/AgCl / V	LUMO level / eV
-1.28	-5.68

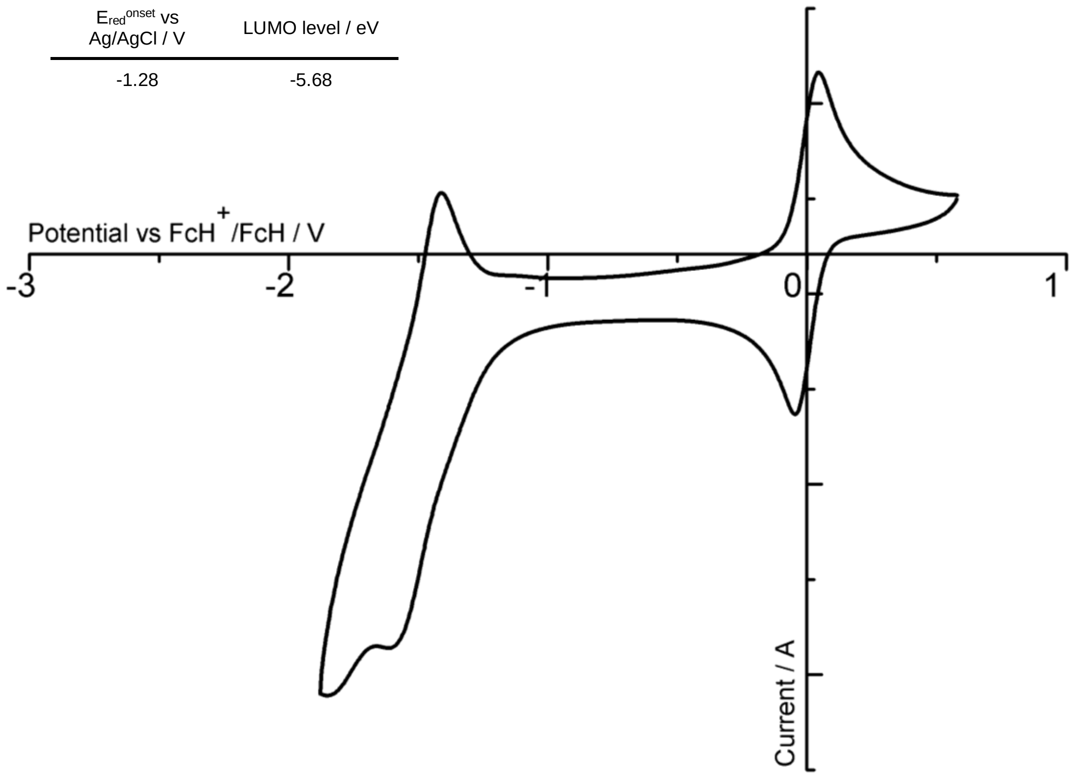

Figure S37. Cyclic voltammogram of **2** in with 0.1 M NBu_4PF_6 in dry DCM electrolyte with a platinum working electrode, Pt counter electrode and an Ag/AgCl (Ag/Ag^+) reference electrode with ferrocene as internal reference.

References

[1] T.-S. Ahn, R. O. Al-Kaysi, A. M. Müller, K. M. Wentz, C. J. Bardeen, *Rev. Sci. Instrum.* **2007**, *78*, 086105.
[2] Gaussian 09, Revision A.02, M. J. Frisch, G. W. Trucks, H. B. Schlegel, G. E. Scuseria, M. A. Robb, J. R. Cheeseman, G. Scalmani, V. Barone, G. A. Petersson, H. Nakatsuji, X. Li, M. Caricato, A. Marenich, J. Bloino, B. G. Janesko, R. Gomperts, B. Mennucci, H. P. Hratchian, J. V. Ortiz, A. F. Izmaylov, J. L. Sonnenberg, D. Williams-Young, F. Ding, F. Lipparini, F. Egidi, J. Goings, B. Peng, A. Petrone, T. Henderson, D. Ranasinghe, V. G. Zakrzewski, J. Gao, N. Rega, G. Zheng, W. Liang, M. Hada, M. Ehara, K. Toyota, R. Fukuda, J. Hasegawa, M. Ishida, T. Nakajima, Y. Honda, O. Kitao, H. Nakai, T. Vreven, K. Throssell, J. A. Montgomery, Jr., J. E. Peralta, F. Ogliaro, M. Bearpark, J. J. Heyd, E. Brothers, K. N. Kudin, V. N. Staroverov, T. Keith, R. Kobayashi, J. Normand, K. Raghavachari, A. Rendell, J. C. Burant, S. S. Iyengar, J. Tomasi, M. Cossi, J. M. Millam, M. Klene, C. Adamo, R. Cammi, J. W. Ochterski, R. L. Martin, K. Morokuma, O. Farkas, J. B. Foresman, and D. J. Fox, Gaussian, Inc., Wallingford CT, 2016., *Gaussian 09, Revision D.01*, **n.d.**
[3] T. Körzdörfer, J.-L. Brédas, *Acc. Chem. Res.* **2014**, *47*, 3284–3291.
[4] M. Little, The Synthesis of Novel Polycyclic Aromatic Hydrocarbons: The Search for Organic Semiconductor Materials, University of Manchester, **2014**.
[5] Y. Tobe, S. Saiki, N. Utsumi, T. Kusumoto, H. Ishii, K. Kakiuchi, K. Kobiro, K. Naemura, *J. Am. Chem. Soc.* **1996**, *118*, 9488–9497.
[6] H. Naito, Y. Morisaki, Y. Chujo, *Angew. Chem. Int. Ed.* **2015**, *54*, 5084–5087.
[7] H. Naito, K. Nishino, Y. Morisaki, K. Tanaka, Y. Chujo, *Chem. – Asian J.* **2017**, *12*, 2134–2138.
[8] H. Naito, K. Nishino, Y. Morisaki, K. Tanaka, Y. Chujo, *J. Mater. Chem. C* **2017**, *5*, 10047–10054.
[9] H. Naito, K. Nishino, Y. Morisaki, K. Tanaka, Y. Chujo, *Angew. Chem. Int. Ed.* **2017**, *56*, 254–259.
[10] E. Lippert, *Electrochemistry* **1957**, *61*, 962–975.
[11] C. M. Hansen, *Hansen Solubility Parameters: A User's Handbook, Second Edition*, CRC Press, Boca Raton, **2007**.
[12] N. M. O'boyle, A. L. Tenderholt, K. M. Langner, *J. Comput. Chem.* **2008**, *29*, 839–845.

Author Contributions

Adam V. Marsh – Lead investigator, data curator and writer of original draft.
Nathan J. Cheetham – Supporting, all optical data acquisition and analysis.
Mark Little – Supporting, synthesis.
Matthew Dyson – Supporting, low-temperature PL data acquisition and analysis.
Andrew J. P. White – Supporting, XRD data acquisition and analysis.
Peter Beavis – Supporting, funding acquisition, general contribution.
Paul N. Stavrinou – Supporting, optical data analysis.
Martin Heeney – Principle investigator.
